# Supplementary material for: Unravelling the Role of Uncommon Hydrogen Bonds in Cyclodextrin Ferrociphenol Supramolecular Complexes: A Computational Modelling and Experimental Study
Source: Int J Mol Sci. 2023 Jul 31;24(15):12288. doi: 10.3390/ijms241512288 (PMC10419020; doi:10.3390/ijms241512288)
Supplement: Supplementary file 1 [file ijms-24-12288-s001.zip › ijms-2515428-supplementary.pdf]

Supporting information

Unravelling the role of uncommon hydrogen bonds in cyclodextrin ferrociphenol supramolecular complexes: a computational modelling and experimental study

Pascal Pigeon [1,2,]*, Feten Najlaoui [3], Michael J. McGlinchey [4], Juan Sanz García [5], Gérard Jaouen [1,2] and Stéphane Gibaud [3]

[1] PSL, Chimie ParisTech, 11 rue Pierre et Marie Curie, Paris F75231 Paris cedex 05, France
[2] Sorbonne Université, Institut Parisien de Chimie Moléculaire (IPCM) – UMR 8232, 4 place Jussieu, 75252 Paris Cedex 05, France; pascal.pigeon@sorbonne-universite.fr
[3] Université de Lorraine, EA 3452/CITHEFOR, 5 rue Albert Lebrun (Faculté de Pharmacie), F-54000 Nancy, France
[4] UCD School of Chemistry, University College Dublin Belfield, Dublin 4, Ireland
[5] 1MSME, Université Gustave Eiffel, CNRS, 5 Boulevard Descartes 77454 Marne-la-Vallée, France

Links:

All files needed for this work can be found into the data repository at https://doi.org/10.57745/CBUPP3. This includes the PHP program, the C program, the database, the static webpages, and other files (follow the link above and read the README.txt file for more details).

The PHP program (named CDModelTree) was also deposited into a forge (Software Heritage) using Hal:

Pascal Pigeon. CDModelTree (V1): Manipulation of a cyclodextrin-ferrociphenol supramolecular models database using a PHP web application. 2022, ⟨swh:1:dir:5c05be4fc1a1a2d4ca16bc48323231bb28379dec;origin=https://hal.archives-ouvertes.fr/hal-03991394;visit=swh:1:snp:438a3325327d8bef3a099271997a0dbdeca32da2;anchor=swh:1:rel:4ec1d7207a58cc63963137f2e79030f04cfdec96;path=/⟩. ⟨hal-03991394⟩

SUMMARY: **page**

Modelling (tables S1 and S2)	3
Classification trees for models of SuccFerr-βCD assemblies (Fig. S1-S2)	7
Web application	16
Database and SQL	18
The PHP program and the webpages (table S3)	27
Installation of XAMPP, of the database and of the PHP software	29
Useful algorithms (Fig. S3)	30
Case of 1-CD series 9 and 10	39
Figure S4. Algorithm of the two PHP programmes	41
Examples without XAMPP (static webpages)	42
C program to control the models	42
Case of the best models of 1CD series 8 and series 2 (fig. S5-S6)	49
**Table S4.** Second-order perturbation stabilization energies for the NBOs	51
Description of the trees of modelling and of the web application to manage them	54

Modelling

Molecular Modelling calculations with the semiempirical PM3 quantum-mechanical method were performed using the 64-bit program Spartan 14 version 1.1.8 (Wavefunction Co., Irvine, CA, USA) with a Dell Precision T5810 PC (equipped with an Intel® Xeon® CPU E5-1630 v3 at 3.70 GHz, 4 cores, 8 logical CPU, 16 Gb RAM memory, 64 bits Windows 10). DFT calculations were performed using Spartan 20 on a workstation or Gaussian 09 on the supercomputer MeSU at Sorbonne Université, Paris (https://sacado.sorbonne-universite.fr/mesu/).

Each time a model was modified, a geometry optimization was carried out using the Merck molecular force field (MMFF) method followed by the semiempirical PM3 quantum-mechanical method. The semiempirical PM3 quantum-mechanical method was also used to determine the affinity of SuccFerr with the CD. This allowed one to evaluate the affinity of each of the 4 substituents of the alkene double bond for each side of each CD. In this SI, wdMβCD is an abbreviation of well-defined Methyl βCD (all glucose units are identical).

Typical method for inclusion of SuccFerr into well-defined βCDs

Each of the six tested CDs (mono-methylated: 2-Me-βCD, 3-Me-βCD, 6-Me-βCD and dimethylated: 2,3-diMe-βCD, 2,6-diMe-βCD and 3,6-diMe-βCD) were built separately and then optimized as described above. SuccFerr and the CD were copied and pasted into the same new window with forced inclusion of one of the four moieties of the double bond of SuccFerr approximately on the axis of the CD. Each moiety of the SuccFerr molecule, i.e., ferrocenyl (Fc), succinimidylpropyl (Succ), phenol #1 -*cis* with the ferrocenyl (Ph1) - and phenol #2 (Ph2) was included on each side of the CD (wide and narrow side) and built in separated files.

Typical method for inclusion of SuccFerr into two well-defined βCDs

To limit the number of possibilities the two wdMβCDs were identical (the six previous wdMβCDs, even for the special experiment CDxCD that gave two identical CDs as the best G0, see below) and only four combinations were calculated (Fc-Ph_1, Fc-Ph_2, Fc-Succ, Ph_1-Succ). However, the four possible combinations of inclusion were calculated (wide side – wide side, narrow side – narrow side, wide side – narrow side, narrow side – wide side). Files of previous experiments with one CD (CD1) on Fc or Ph_1 were duplicated, and in each copy, a second CD (CD2) was inserted

by copying and pasting the CD1 and placing by forced inclusion one of the remaining 3 substituents (Ph1, Ph2 or Succ for Fc and Succ for Ph1) with an eventual flipping (wide side or narrow side). The affinity required calculation of the energies of the reactions: SuccFerr + CD1 + CD2 → molecular assembly ($\Delta E = E_{SuccFerr\text{-}CD1\text{-}CD2} – E_{CD1} – E_{CD2} - E_{SuccFerr}$).

Table S1. Calculated 1-CD systems (series S1 to S8) for inclusion into narrow or wide side of each of the moieties of SuccFerr into well-defined CDs with monomethylated CDs (2-Me-βCD, 3-Me-βCD, 6-Me-βCD) and dimethylated CDs (2,3-diMe-βCD, 2,6-diMe-βCD, 3,6-diMe-βCD). ΔE in kJ/mol.

**Series 1, narrow side, Fc** [1]	**ΔE**	**Series 2, wide side, Fc**	**ΔE**
**2-Me-βCD** [5]	**- 122**	2-Me-βCD	- 58
3-Me-βCD	- 97	**3-Me-βCD**	**- 83**
6-Me-βCD	- 76	6-Me-βCD	- 43
2,3-diMe-βCD	- 96	2,3-diMe-βCD	- 53
2,6-diMe-βCD	- 73	2,6-diMe-βCD	- 52
3,6-diMe-βCD	- 68	3,6-diMe-βCD	- 75
**Series 3, narrow side, Ph1** [2]	**ΔE**	**Series 4, wide side, Ph1**	**ΔE**
**2-Me-βCD**	**- 111**	2-Me-βCD	- 52
3-Me-βCD	- 34	3-Me-βCD	- 47
6-Me-βCD	- 46	6-Me-βCD	- 53
2,3-diMe-βCD	- 42	2,3-diMe-βCD	- 41
2,6-diMe-βCD	- 72	2,6-diMe-βCD	- 40
3,6-diMe-βCD	- 38	**3,6-diMe-βCD**	**- 141**
**Series 5, narrow side, Ph2** [3]	**ΔE**	**Series 6, wide side, Ph2**	**ΔE**
**2-Me-βCD**	**- 48**	2-Me-βCD	- 37

3-Me-βCD	- 36	3-Me-βCD	- 44
6-Me-βCD	- 21	**6-Me-βCD**	**- 49**
2,3-diMe-βCD	- 34	2,3-diMe-βCD	- 40
2,6-diMe-βCD	- 38	2,6-diMe-βCD	- 28
3,6-diMe-βCD	- 43	3,6-diMe-βCD	- 31
**Series 7, narrow side, Succ [4]**	**ΔE**	**Series 8, wide side, Succ**	**ΔE**
2-Me-βCD	- 61	2-Me-βCD	- 53
3-Me-βCD	- 40	3-Me-βCD	- 81
6-Me-βCD	- 41	6-Me-βCD	- 50
**2,3-diMe-βCD**	**- 161**	2,3-diMe-βCD	- 51
2,6-diMe-βCD	- 74	2,6-diMe-βCD	- 40
3,6-diMe-βCD	- 45	**3,6-diMe-βCD**	**- 111**

[1] Fc: ferrocene, [2] Ph1: phenol 1 (*cis* to Fc), [3] Ph2: phenol 2 (*trans* to Fc), [4] Succ: succinimidylpropyl, [5] The best system and value for each series is displayed in bold and is the starting systems (G0) for calculation for diversely methylated cyclodextrins calculations.

Table S2. Calculated 2-CD systems: Inclusion of 2 moieties of SuccFerr into the wide side (WS) or into the narrow side (NS) of monomethylated wdMβCDs (2-Me-βCD, 3-Me-βCD, 6-Me-βCD) or dimethylated wdMβCDs (2,3-diMe-βCD, 2,6-diMe-βCD, 3,6-diMe-βCD). ΔE in kJ/mol.

Monomethylated			Dimethylated		
2-Me-βCD		ΔE	2,3-diMe-βCD		ΔE
	Fc(WS)+Ph1(WS) [1]	- 132		Fc(WS)+Ph1(WS)	- 104
	Fc(WS)+Ph2(WS)	- 150		Fc(WS)+Ph2(WS)	- 122
	Fc(WS)+succ(WS)	- 118		Fc(WS)+succ(WS)	- 112
	Ph1(WS)+succ(WS)	- 116		Ph1(WS)+succ(WS)	- 93

	Fc(NS)+Ph1(NS)	- 176		Fc(NS)+Ph1(NS)	- 163
	Fc(NS)+Ph2(NS)	- 148		Fc(NS)+Ph2(NS)	- 99
	Fc(NS)+succ(NS)	- 182		Fc(NS)+succ(NS)	- 150
	Ph1(NS)+succ(NS)	- 142		Ph1(NS)+succ(NS)	- 179
	Fc(WS)+Ph1(NS)	- 113		Fc(WS)+Ph1(NS)	- 109
	Fc(WS)+Ph2(NS)	- 126		Fc(WS)+Ph2(NS)	- 90
	Fc(WS)+succ(NS)	- 114		Fc(WS)+succ(NS)	- 99
	**Ph1(WS)+succ(NS)**	**- 246** [2]		Ph1(WS)+succ(NS)	- 120
	Fc(NS)+Ph1(WS)	- 150		**Fc(NS)+Ph1(WS)**	**- 203**
	Fc(NS)+Ph2(WS)	- 162		Fc(NS)+Ph2(WS)	- 173
	Fc(NS)+succ(WS)	- 184		Fc(NS)+succ(WS)	- 78
	Ph1(NS)+succ(WS)	- 178		Ph1(NS)+succ(WS)	- 77
3-Me-βCD		ΔE	2,6-diMe-βCD		ΔE
	Fc(WS)+Ph1(WS)	- 131		Fc(WS)+Ph1(WS)	- 100
	Fc(WS)+Ph2(WS)	- 140		Fc(WS)+Ph2(WS)	- 97
	Fc(WS)+succ(WS)	- 111		Fc(WS)+succ(WS)	- 77
	Ph1(WS)+succ(WS)	- 96		Ph1(WS)+succ(WS)	- 111
	Fc(NS)+Ph1(NS)	- 150		Fc(NS)+Ph1(NS)	- 112
	Fc(NS)+Ph2(NS)	- 187		Fc(NS)+Ph2(NS)	- 82
	Fc(NS)+succ(NS)	- 89		Fc(NS)+succ(NS)	- 79
	Ph1(NS)+succ(NS)	- 145		Ph1(NS)+succ(NS)	- 89
	Fc(WS)+Ph1(NS)	- 122		Fc(WS)+Ph1(NS)	- 87
	Fc(WS)+Ph2(NS)	- 110		Fc(WS)+Ph2(NS)	- 70
	Fc(WS)+succ(NS)	- 124		Fc(WS)+succ(NS)	- 84
	Ph1(WS)+succ(NS)	- 132		Ph1(WS)+succ(NS)	- 87
	Fc(NS)+Ph1(WS)	- 133		Fc(NS)+Ph1(WS)	- 114
	**Fc(NS)+Ph2(WS)**	**- 208**		Fc(NS)+Ph2(WS)	- 118
	**Fc(NS)+succ(WS)**	**- 278**		Fc(NS)+succ(WS)	- 106
	Ph1(NS)+succ(WS)	- 76		Ph1(NS)+succ(WS)	- 112
6-Me-βCD		ΔE	3,6-diMe-βCD		ΔE

Fc(WS)+Ph1(WS)	- 141	Fc(WS)+Ph1(WS)	- 118
Fc(WS)+Ph2(WS)	- 119	Fc(WS)+Ph2(WS)	- 135
Fc(WS)+succ(WS)	- 116	Fc(WS)+succ(WS)	- 118
Ph1(WS)+succ(WS)	- 129	Ph1(WS)+succ(WS)	- 104
Fc(NS)+Ph1(NS)	- 130	Fc(NS)+Ph1(NS)	- 129
Fc(NS)+Ph2(NS)	- 143	Fc(NS)+Ph2(NS)	- 94
Fc(NS)+succ(NS)	- 120	Fc(NS)+succ(NS)	- 91
Ph1(NS)+succ(NS)	- 103	Ph1(NS)+succ(NS)	- 94
Fc(WS)+Ph1(NS)	- 131	Fc(WS)+Ph1(NS)	- 78
Fc(WS)+Ph2(NS)	- 87	Fc(WS)+Ph2(NS)	- 108
Fc(WS)+succ(NS)	- 93	Fc(WS)+succ(NS)	- 101
Ph1(WS)+succ(NS)	- 123	Ph1(WS)+succ(NS)	- 73
Fc(NS)+Ph1(WS)	- 143	Fc(NS)+Ph1(WS)	- 108
Fc(NS)+Ph2(WS)	- 170	Fc(NS)+Ph2(WS)	- 123
Fc(NS)+succ(WS)	- 106	Fc(NS)+succ(WS)	- 98
Ph1(NS)+succ(WS)	- 119	Ph1(NS)+succ(WS)	- 87

[1] Fc: ferrocene, Ph1: phenol 1 (*cis* to Fc), Ph2: phenol 2 (*trans* to Fc), Succ: succinimidylpropyl. Calculated combinations are WS-WS, NS-NS, WS-NS or NS-WS, but excluding all Ph1-Ph2 and Ph2-Succ combinations.[2] The best system and value for each series is displayed in bold and is the starting systems (G0) for calculation for diversely methylated cyclodextrins calculations.

Classification trees for models of SuccFerr-βCD assemblies

General background:

The information related to our SuccFerr-cyclodextrin complex models generate a large amount of data that needs to be saved reliably, but also to be readily consulted and even used in statistics or other calculations. The ideal method is therefore to save the data in a database which makes it possible to retrieve the desired information from thousands of other records by sorting and selecting using simple queries. The free pack XAMPP (version 8.2.0 was used) can be used to manage this very large scientific database. It contains a web server program (Apache), a database (MariaDB) supporting the SQL query language, a web system for manually entering SQL queries (phpMyAdmin) and a programming language interpreter (PHP). Access to the server through a web browser (whichever one is used, and whatever the computer's operating system) allows

information to be entered and displayed in HTML format, but also to make decisions automatically (PHP program). In addition to this database, all models are available in XYZ format satisfying the Findability, Accessibility, Interoperability and Reusability (FAIR) approach and additional treatments and statistics on them were carried out using a handmade C program. Together with other recognized technologies, such as ioChem-BD, AiiDa, ASE or Flask, these new packages can help in the exploitation and management of large amounts of computational data. More details follow.

Method:

In this work, we show how our web application was used to classify a series of cyclodextrin models (here limited to βCDs) according to their methylation state. These cyclodextrins serve to solubilize SuccFerr (Figure 2 of main text), which is very active against cancer cells but poorly soluble in water.

We have used the semiempirical PM3 quantum-mechanical molecular modelling method (abbreviated as “PM3”) to determine which methylated βcyclodextrins are the ablest to make a stable supramolecular assembly in order to solubilize SuccFerr. We calculated the models of SuccFerr with one or two cyclodextrins (1-CD or 2-CD systems), using different types of βCD. Assembly with 3 CDs was ruled out early on as models showed poor inclusion of “moieties” in the CD cavity, and instead the formation of aggregate-like assemblies. Considering the central double bond of SuccFerr, we can envisage four molecular fragments, designated here as "moieties" (shown in Figure 2 of main text, surrounded by purple rectangles) likely to enter the CD cavity: the ferrocenyl group (Fc), phenol 1 (Ph1, *cis* to the Fc), phenol 2 (Ph2, *trans* to the Fc) and the succinimidylpropyl group (Succ). Each moiety can access the cavity of the CD either from the wide side or from the narrow side. For 1 CD systems, this gives a total of 8 possible combinations, which we label as 1-CD series 1 to 8. For 2-CD systems, there are 24 possible series (6 combinations of 2 moieties times 4 combinations of wide and narrow sides). When one of these four moieties of SuccFerr is encapsulated within a CD, all 21 oxygen atoms become non-equivalent. We have therefore numbered (O1 to O21) each of the oxygen atoms that can be (de)methylated in order to identify them unambiguously not only in our models, but also in our database (Figure 1 of main text). A specific structure can be found in this database using a complex SQL query with the appropriate index for each oxygen atom (value of each Oi is even numbers for unmethylated oxygen atoms and odd numbers for methylated ones). Based on the glucose numbering system, oxygens at position 2 are given a number 3n + 1, those at the positions 3 are

listed as 3n + 2, and those at positions 6 are labelled as 3n + 3, with n ($0 \leq n \leq 6$) being the anhydroglucose unit number. For simplicity, this number is not referenced in this article, and the first unit (n = 0) was chosen arbitrarily for each series. The SQL query to search for all supramolecular assemblies involving a specific CD, referred as “free CD”, is simpler and consists of finding all records containing a certain number. This number is a binary conversion of the CD configuration (unmethylated = 0, methylated = 1), retaining the smaller of the seven possible numbers from now on “binarymin” (depending on the starting anhydroglucose unit number, see details in SI: “Algorithm to calculate binarymin” and Figure S3). The number of possible systems for each of these 8 series becomes 2^{21} = 2,097,152 combinations (2^{42} for 2-CD systems); evidently, these are impossible to calculate with limited resources in a reasonable time. Thus, we have devised a simple, robust, and sound algorithm to reduce the number of combinations to be computed (see “undefined-CD” section). This algorithm is based on the stability of the CD-SuccFerr complex. The computed stability requires calculation of the energies of the SuccFerr-CD(s) molecular assembly, of each CD, and of SuccFerr, the latter two in the conformations they had in the molecular assemblies to give the ΔE (energy variations, equation 2 or 3) of the reactions: SuccFerr + CD_1 [+ CD_2] → molecular assembly. Note that equation 3 also takes into account the eventual interactions between the two CDs, which makes it possible to predict a more negative ΔE than that of 1-CD systems.

Eq. for 1-CD: $\Delta E = E_{(SuccFerr+CD)} - E_{SuccFerr} - E_{CD1}$

Eq. for 2-CD: $\Delta E = E_{(SuccFerr+CD1+CD2)} - E_{SuccFerr} - E_{CD1} - E_{CD2}$

We started with well-defined CDs (see definition above) as a basic model, in order not only to compare them with undefined CDs but also to create the latter by modifying the states of methylation.

- Well-defined CDs; Having no idea which types of methylation would work best for each combination, the 8 1-CD series were each comprised of 6 well-defined CDs: three mono-methylated combinations: 2-Me-βCD, 3-Me-βCD, and 6-Me-βCD (i.e., on all the oxygen atoms at positions 2, 3 or 6, respectively, of their 7 anhydroglucose units, i.e., all glucose units are identical), and three dimethylated combinations: 2,3-diMe-βCD, 2,6-diMe-βCD (DMβCD) and 3,6-diMe-βCD (Table S1). The same calculations were made with the 2-CD assemblies (reduced to 16 of the 24 possible, eliminating models with bad inclusion characteristics, Table S2).

- Undefined CDs; The well-defined CDs were then used as the basic model on which to create the undefined CDs, that is, those with any combination of methylations. First, so as to start from already relatively stable models, the best one from each of the 8 series of the previous 1-CD models (i.e., having the most negative ΔE) was chosen (Table S1). For the 2-CD models, only the 4 best series (2-CD series 1 to 4, taken from Table S2) were studied because calculation times would be too long. When creating methylation modification trees (resembling a tree of DNA / RNA / protein mutations but without the deletions and insertions), these 12 basic systems were classified as generation zero (G0). By analogy, we will use the term "mutation" for each change in the methylation pattern, even if this nomenclature is perhaps non-standard. Initially, we are only allowed to continue mutations into the next generation (Gn + 2) if ΔE has decreased between Gn and Gn + 1 (rule 1). The series 2-CD S4 was exempted of this rule 1 to analyze its effect and utility (the remaining very best model without descendant of the whole series was systematically used to continue in the next generation, even if its ΔE has increased). Of course, it is only interesting when this model has a ΔE more negative than that of any model with a decreasing ΔE.

Rule 1: (applied to any series but 2-CD S4)
If(ΔE(Gn+1) < ΔE(Gn)) then

Allow to continue with descendants Gn+2 within this branch (creation of new ramifications, one for all descendants)

else

stop (creation of new ramifications Gn + 2 are not allowed starting from this experiment of generation Gn + 1)

For the 8 series 1-CD and 4 series 2-CD, 21 descendants of G0 (42 for 2-CD systems) were created, each corresponding to a reversal of the methylation of the G0 system onto a different oxygen atom, creating the first generation of mutations (G1). The best blend (most negative ΔE) of the 21/42 G1 offspring in each series was in turn used as a template for the creation of a new mutation (G2). At the start of the branch, the first mutations (G1, G2, G3, etc.) were retained: rule 2: no modification of the methylation of an oxygen atom several times in the same chain / route of modifications. The series 2-CD S4 was also exempted of this rule 2 for the same purpose of studying the effect of this rule.

Rule 2: (applied to any series but 2-CD S4)

nbOH = 21 (1-CD models) or 42 (2-CD models)

for (i = 1; i ≤ nbOH; i++) ← outer loop going through all oxygen atoms of current system, of generation Gn, to be modified

    for (j = 1; j < Gn; j++) ← inner loop going through all previous generations Gj of this route, starting from G1

        if(O_i of ancestor Gj was modified) then

        mark O_i as non-modifiable (will be ignored)

    if(O_i is not marked) then

        allow inversion of methylation

The goal of fixing these mutations is a logical choice: If they have had a positive effect, it is advisable not to cancel them in the following mutations, or in any case not soon after. However, when many methylations have been altered, the complex interaction can cause a mutation that was useful early on in advancing the stability of the SuccFerr-CD assembly, only to become troublesome later on. This has been verified several times in this study. In this case it is necessary, at a certain stage, mainly at the end of the road, to authorize this mutation at will (bypass of rule 2). In this study, the "end of the road" (dead end) will be how we describe a chain of modifications leading to a generation where none of its systems have seen their ΔE drop, which leads to a dead end (locally, a model not allowed by rule 1 is also a type of dead end). Bypass of rule 2 is applied to a certain parent experiment chosen by the user and concerns only its direct descendants Gn + 1, without changing the mode of operation of the rest of the tree. It brings up, for this parent experiment, other descendants to be calculated, which can unblock the road for a few more generations, as has been verified in this work. However, when even the bypass of rule 2 cannot prevent the end of the route, it may also be interesting to remove rule 1 (bypass of rule 1), although this only seems to make sense for better systems whatever branch they are in, and not only the current one. The exception is 2-CD S4 where rules 1 and 2 were not applied from the start, as stated above, for comparison of the two methods (with or without rules). Indeed, with a strict application of rule 1, a good system, but going through a bad stage, would never be reached, or by making a big detour. For example, the very best system # 3029 (unique value of its key 'ID') belonging to series 4 (the only series where rule 2 is systematically bypassed) of 2-CD systems, has undergone a chain of mutations starting from the G0 system: G0 - O_{37} + O_2 - O_{31} - O_{40} + O_{18} -

O_4 - O_2 + O_{36} - O_{16} + O_{48} + O_{44} - O_{43} - O_{36} - O_{44} + O_{42} + O_{16} - O_7 + O_{44} - O_{42} + O_{36} - O_{48} + O_2 - O_1 + O_7 + O_{48} - O_{19} (plus = methylation, minus = demethylation, of the indicated oxygen atom). The demethylation of O_{48}, carried out in G25 (ID = 2029), produced a rise of ΔE, and without the bypass of rule 1, this chain, which continues until G27 (end of the route), would have been blocked in G25. Likewise, we see a demethylation of O_{44} carried out in G14 (ID = 2630), while this atom had already been modified in G11 (ID = 2518), and that without bypassing rule 2, the chain would have stopped in G14. Moreover, we also see that this atom is modified a third time in G18, just like O_{48} (G10, G21 et G25) and O_{36} (G8, G13 et G20). This latest information confirms what was previously described, namely that a change, and its undoing a few generations later, can both lead to an improvement in the system.

Always, in order to find more good systems, it is unavoidable to create bifurcations (creation of a tree of mutations) since a single road will necessarily ends, even with bypasses. Going back to generations before the end of the road and creating a fork from the second / third best system respecting rule 1 creates parallel chains and therefore the branches of this tree of mutations. It should be noted that this does not apply to 2-CD S4, where the current very best system is chosen, respecting rules 1, rule 2 or none, being inside the current road or inside a parallel one. Some of these parallel chains can lead to the same configuration (same mutations but in a different order), which would create a duplicate in the tree which would no longer be a spanning tree. To avoid this, you need a system that checks, before creating an experiment, that there is no risk of producing a duplicate by checking whether it already exists in the database (rule 3: prohibit duplicates). This rule 3 is thus systematically applied in each road to avoid doing a mutation in Gn + 2 that would lead to the same model Gn, by cancelling the mutation done in Gn +1.

Rule 3: (no bypass allowed)

```
for (i = 1; i ≤ All structures this series; i++)
        if(Structure_i already exists), then
                Structure_i is displayed for information
        else
                Structure_i is allowed to be created in database
```

To summarize, to determine if rules 1 and 2 are really useful, we applied two methods and compared their effect:

- Method I; For all 1-CD series and for the first three 2-CD series, we apply rules 1 and 2 at the beginning of the trees and decide to lift these rules (bypass) when the progression becomes slower (the number of dead ends becomes significant). This method therefore requires the user to decide on when to apply these bypasses. We must then decide to bypass rule 2, but also decide if we do it with, or without, simultaneously bypassing rule 1. This method is therefore less clear;
- Method II; For the 2-CD S4 series, we systematically bypass rules 1 and 2 from the start (G0). The user therefore follows the algorithm without having to decide. Finally, to produce this 2-CD S4 series, a final adjustment was made: To simplify, for the 2-CD S1 to S3 series, we took the same CD for both insertions. But there was no reason for this CD to be the best one for both moieties at the same time, and therefore for us to start from the model with the lowest possible ΔE (using the best model of Table S2, reported in Table 1). Therefore, we made the 6 x 6 possible combinations, with the help of a new PHP program (CDxCD.php, provided) and a new database table (CDxCD, provided) and chose the best model as G0 of our tree for S4. Ironically, a model with twice the same CD (2-Me-CD) gave the best result!

Of course, rule 3 remains mandatory for both methods.
Figure S1 shows examples on a fictitious tree and Figure S2 compares the computer flowchart of methods I and II:

1. Rule 1: The mutations indicated all correspond to decreases in ΔE except the G0 + O_4 + O_6 route where methylation on oxygen atom 6 causes an increase in ΔE and will not be allowed to continue in G3 (bypass possible if the user so desires, and preferably only when reaching the ends of the road leading to the best systems or if this model is the current very best for 2-CD S4);
2. Rule 2: The mutation (G0 + O_4 - O_8 + O_5 - O_4 modifies oxygen atom 4 a second time and will not be authorized (but bypass possible, especially useful at the end of the road). For 2-CD S4, bypass is systematic;
3. Rule 3: The G0 + O_4 - O_8 + O_5 - O_9 - O_3 mutation gives the same configuration as the G0 + O_4 - O_8 - O_3 + O_5 - O_9 route, so the -3 mutation will be blocked, and this route will stop on demethylation in 9 (bypass not authorized whatever is the series).

The simplified computer flowchart common to the two PHP programs which manages the trees of the 1-CD and 2-CD system is shown in Figure S4.

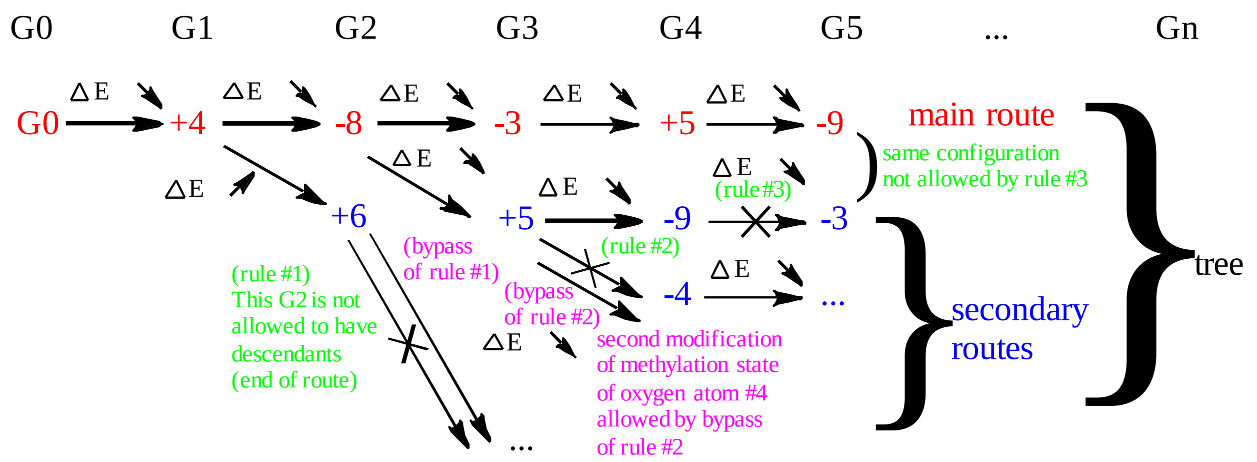

Figure S1. Example of a fictitious mutation tree with explanation of the rules. + indicates methylation on the oxygen atom of the number indicated and - demethylation. The main route/road is the one that goes through the best system of each generation and the secondary roads those that go through at least one system that is not the best of its generation. G0 (generation 0) represents the well-defined starting CD and G1, G2,… the following generations.

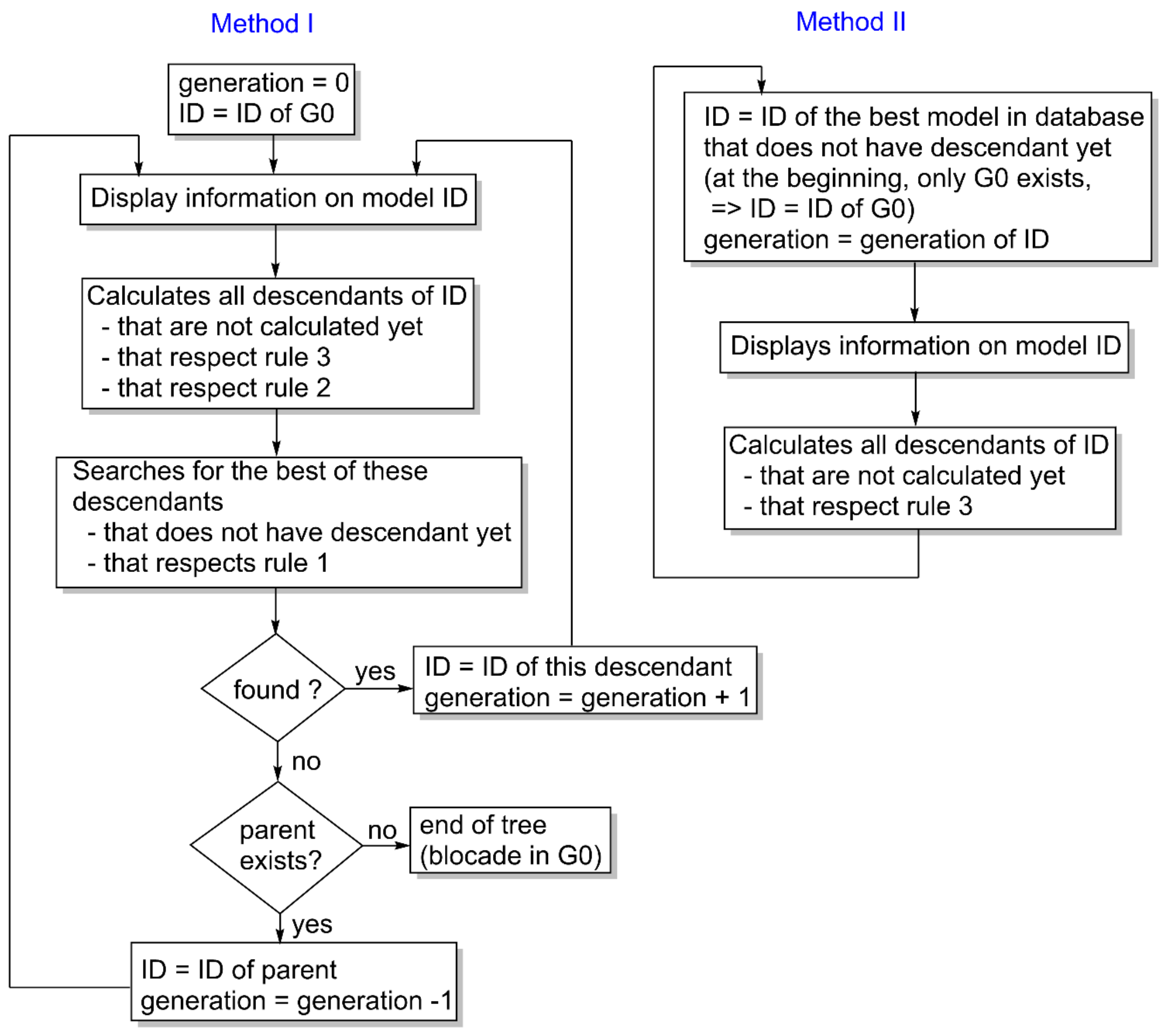

Figure S2. computer flowchart describing method I (left: rules 1 and 2 are respected) and method II (right: rule 1 and 2 are waived).

Comparing methods I and II

The study of the paths of mutations ('path' fields of the database) shows that with the bypass of rule 2, certain mutations are successfully cancelled sometimes only three generations later (for example, ID 7692 and 7759 for O16). We can therefore ask ourselves the question of the validity of rule 2, especially since its bypass is chosen more or less arbitrarily by the user, which introduces serendipity into the calculations and therefore confusion into the case where we want to apply this method for other systems. Similarly, rule 1 seems counter-productive. Indeed, when we arrive at an end of route in Gn, the method I that we used consists in downgrading by taking the second-best model respecting rule 1 of the previous generation (in Gn-1, if this model exists otherwise in Gn-2, … see computer flowchart Figure S2 method I). This prolongs a parallel branch (a bifurcation with just one model up to now) in the tree, whereas a model not respecting this rule, in the current generation (Gn) may have a better affinity and not lead to the creation of a long new branch but to the extension of the current branch (tree less ramified and therefore less confusing). Method II not applying these two rules 1 and 2 with the 2-CD S4 series gives good results, but with a larger number of calculations (Figure S2 method II). This new simplified method II is therefore to be preferred over the old method I, even with more calculations (that also could lead to better models by serendipity).

Web application

To help handle the considerable number of experiments (9,767 for 1-CD calculations and 3,458 for 2-CD calculations) and corresponding data created by the ten series of experiments for 1-CD calculations and four series for 2-CD calculations, and to verify and respect the rules explained in section “Undefined CD” in the main text, an application was specially created with the XAMPP pack (PHP, MySQL/MariaDB and phpMyAdmin) (https://www.apachefriends.org). The pack should be installed (Windows, Linux, or OS X) to run the application and operate the database (see below).

Alternatively, to consult the results that are displayed into the dynamic webpages by the PHP program, static webpages, one for each model, were created by the C program (see

below). This C program permitted to avoid to manually save from the browser all pages one by one. The links operate off-line without XAMPP (they were adapted), but not the forms.

Description of the system:

More details can be found at https://hal.science/hal-03991394 (then follow the link towards Software Heritage or directly use the link displayed in page 1 of this document).

- The data were stored into a database named 'pascal' operated by XAMPP (MariaDB, database engine: MyISAM, one table (calculscd) for the eight series of 1-CD calculations (+ 2 extra series S9 and S10, see below), one table (calculs2cd) for the four series of 2-CD calculations) and one table (cdxcd) for the special experiment to find the best model used as G0 for series 2-CD S4 (see above).
- Starting G0 systems for each series were either manually entered the table of the database using the phpMyAdmin software or simply created by a special option in the software by entering the methylation state of position 2, 3 and 6 for the seven anhydroglucose units. The other experiments, derived from them, were stored by the same software.
- The software was coded in PHP and was stored into three files (cdmodele.php to handle 1-CD calculations, cdmodele2cd.php for 2-CD calculations and CDxCD.php for the special experiment to find the best G0 for 2-CD S4, see below). It allows one to:
    - create a dynamic web page displaying the forms and the data of the trees into a browser (location: http://localhost/cdmodele.php for 1-CD calculations, http://localhost/cdmodele2cd.php for 2-CD calculations and http://localhost/CDxCD.php?action=show for special experiment). If the server (where the XAMPP pack is installed) and the client (where the browser operates) are not the same computer, "localhost" should be replaced by the IP address of the server,
    - receive the data sent from the forms (energies of the supramolecular assembly and of each of its components),
    - calculate ΔE according to the previous formula inside the main text of the article (eq. 2 for cdmodele.php, eq. 3 for cdmodele2cd.php and CDxCD.php),
    - store them into the table,

- allow continuation of experiments in the next generation for ameliorated systems only (rule 1) or force calculation of some next generations (bypass of rule 1 by direct access to them by typing their parent ID number in a form in the webpage),
- create new allowed pending calculations into the database and keep track of them,
- only allow calculations of systems not already treated by another branch (rule 3) and, if rule 2 activated (deactivated by clicking on a link), not corresponding to an already modified oxygen atom in the path from G0 to the present system,
- calculate the statistics and automatically display them when displaying the G0 level of the tree of the current series (see static webpages 1-CD S1 to 1-CD S8 and 2-CD S1 to 2-CD S4 (.html) in compressed archive), display statistics for the eight series of 1-CD calculations into a table for comparison, …

- The web page, used as an interface, allows one to display the forms:
    - buttons for the creation of new pending experiments, if allowed, and direct access to a specific experiment for an eventual bypass of rule 1,
    - fields to input (copy/past from Spartan) calculated energies,
    - hypertext links (navigation forward and backward inside the trees, bypass of rule 2 or change of current series), …

Database and SQL:

The structure of the database (tables calculscd, calculs2cd and cdxcd) is stored into the SQL file ‘pascal.sql’. This file, that contains SQL queries to create the database with its data, should be used to import the database into the MariaDB system using the ‘Import’ option of phpMyAdmin (or other database management systems). This SQL file is the one that should preferably be used to recreate our database identically, because it includes the constraints on the keys, thanks to its system-independent SQL commands and is therefore more portable.

For 1-CD calculations, the unique table for the 10 series inside the database was created with this SQL command:

```
CREATE TABLE `calculscd` (
  `ID` int(16) UNSIGNED NOT NULL,
```

```
  `O1` tinyint(4) NOT NULL,
  `O2` tinyint(4) NOT NULL,
  `O3` tinyint(4) NOT NULL,
  `O4` tinyint(4) NOT NULL,
  `O5` tinyint(4) NOT NULL,
  `O6` tinyint(4) NOT NULL,
  `O7` tinyint(4) NOT NULL,
  `O8` tinyint(4) NOT NULL,
  `O9` tinyint(4) NOT NULL,
  `O10` tinyint(4) NOT NULL,
  `O11` tinyint(4) NOT NULL,
  `O12` tinyint(4) NOT NULL,
  `O13` tinyint(4) NOT NULL,
  `O14` tinyint(4) NOT NULL,
  `O15` tinyint(4) NOT NULL,
  `O16` tinyint(4) NOT NULL,
  `O17` tinyint(4) NOT NULL,
  `O18` tinyint(4) NOT NULL,
  `O19` tinyint(4) NOT NULL,
  `O20` tinyint(4) NOT NULL,
  `O21` tinyint(4) NOT NULL,
  `changed` tinyint(4) NOT NULL,
  `path` text NOT NULL,
  `IDparent` int(16) UNSIGNED NOT NULL,
  `deltaE` float NOT NULL,
  `stop` tinyint(2) NOT NULL,
  `series` smallint(6) NOT NULL,
  `binarymin` int(16) UNSIGNED NOT NULL,
  `generation` tinyint(2) UNSIGNED NOT NULL,
  `deltadelta` float NOT NULL,
  `mainseries` smallint(6) NOT NULL
) ENGINE=MyISAM DEFAULT CHARSET=latin1;
```

For 2-CD calculations, the unique table (calculs2cd) was created with this SQL command:

```sql
CREATE TABLE `calculs2cd` (
  `ID` int(16) UNSIGNED NOT NULL,
  `O1` tinyint(4) NOT NULL,
  `O2` tinyint(4) NOT NULL,
  `O3` tinyint(4) NOT NULL,
  `O4` tinyint(4) NOT NULL,
  `O5` tinyint(4) NOT NULL,
  `O6` tinyint(4) NOT NULL,
  `O7` tinyint(4) NOT NULL,
  `O8` tinyint(4) NOT NULL,
  `O9` tinyint(4) NOT NULL,
  `O10` tinyint(4) NOT NULL,
  `O11` tinyint(4) NOT NULL,
  `O12` tinyint(4) NOT NULL,
  `O13` tinyint(4) NOT NULL,
  `O14` tinyint(4) NOT NULL,
  `O15` tinyint(4) NOT NULL,
  `O16` tinyint(4) NOT NULL,
  `O17` tinyint(4) NOT NULL,
  `O18` tinyint(4) NOT NULL,
  `O19` tinyint(4) NOT NULL,
  `O20` tinyint(4) NOT NULL,
  `O21` tinyint(4) NOT NULL,
  `O31` tinyint(4) NOT NULL,
  `O32` tinyint(4) NOT NULL,
  `O33` tinyint(4) NOT NULL,
  `O34` tinyint(4) NOT NULL,
  `O35` tinyint(4) NOT NULL,
  `O36` tinyint(4) NOT NULL,
  `O37` tinyint(4) NOT NULL,
```

```
  `O38` tinyint(4) NOT NULL,
  `O39` tinyint(4) NOT NULL,
  `O40` tinyint(4) NOT NULL,
  `O41` tinyint(4) NOT NULL,
  `O42` tinyint(4) NOT NULL,
  `O43` tinyint(4) NOT NULL,
  `O44` tinyint(4) NOT NULL,
  `O45` tinyint(4) NOT NULL,
  `O46` tinyint(4) NOT NULL,
  `O47` tinyint(4) NOT NULL,
  `O48` tinyint(4) NOT NULL,
  `O49` tinyint(4) NOT NULL,
  `O50` tinyint(4) NOT NULL,
  `O51` tinyint(4) NOT NULL,
  `changed` tinyint(4) NOT NULL,
  `path` text NOT NULL,
  `IDparent` int(16) UNSIGNED NOT NULL,
  `deltaE` float NOT NULL,
  `stop` tinyint(2) NOT NULL,
  `series` smallint(6) NOT NULL,
  `binarymin1` int(16) UNSIGNED NOT NULL,
  `binarymin2` int(16) UNSIGNED NOT NULL,
  `generation` tinyint(2) UNSIGNED NOT NULL,
  `deltadelta` float NOT NULL,
  `mainseries` smallint(6) NOT NULL
) ENGINE=MyISAM DEFAULT CHARSET=latin1;
```

For the database to respond fast enough to requests, keys/index should be set on fields ‘ID’ (it is mandatory to have unique values for this field, so ‘ID’ was auto incremented by the database engine at each insertion of experiment) and ‘IDparent’ (mandatory for a large set of systems, because the database is slow without it).

These two options are set by the SQL command:

ALTER TABLE `calculscd` ADD UNIQUE KEY ` ID` (`ID`) USING BTREE, ADD KEY `IDparent` (`IDparent`);

For the ‘ID’ field to be set in auto_increment mode:

ALTER TABLE `calculscd` CHANGE `ID` `ID` INT(16) UNSIGNED NOT NULL AUTO_INCREMENT;

The version in the pascal.sql file is a little different from the initial one (creation of an empty table), since it considers the already filled (with 9767 models, the next one to be created being 9768) table:

ALTER TABLE `calculscd` MODIFY `ID` int(16) UNSIGNED NOT NULL AUTO_INCREMENT, AUTO_INCREMENT=9768;

For the calculs2cd table, the SQL commands are:

ALTER TABLE `calculs2cd` ADD UNIQUE KEY ` ID` (`ID`) USING BTREE, ADD KEY `IDparent` (`IDparent`);

ALTER TABLE `calculs2cd` CHANGE `ID` `ID` INT(16) UNSIGNED NOT NULL AUTO_INCREMENT;

Here too, we can see that the table is already filled (with 3458 models, the next one to be created being 3459) table:

ALTER TABLE `calculs2cd` MODIFY `ID` int(16) UNSIGNED NOT NULL AUTO_INCREMENT, AUTO_INCREMENT=3459;

There is no need to create a multicolumn index, as unicity constraint for example. Indeed, the PHP software verifies the constraints (rule 3) before creating experiments. Moreover, it would be impossible, since the Oi fields can take value 0, 1, 2, 3, 6 or 7 (the unicity should be based on the bit 0 of these fields (0 = unmethylated (even number), 1 = methylated(odd number)), not on the value itself.

Examples of useful SQL requests:

Calculation/recovery of some statistics or values by SQL requests for a particular series of experiments for 1-CD systems (some can be used for 2-CD systems, replacing the table ‘calculscd’ by ‘calculs2cd’): The PHP software can use these SQL requests, but they also can be manually

used using phpMyAdmin (in this last case, the variable '$series' should be replaced by 1 to 10 depending on the series we are interested in):

- The best ΔE value of a series: ***SELECT MIN(deltaE) FROM calculscd WHERE series='$series'***
- The total number of calculated systems of a series: ***SELECT COUNT(*) FROM calculscd WHERE series='$series'***
- The total number of changes of methylation state on a certain oxygen atom n (replace $n by: [1..21] for methylation, [-1..-21] for demethylation) for all the experiments of a series: ***SELECT count(*) FROM calculscd WHERE changed='$n' AND series='$series'***
- The same, but only changes that improved ΔE (respecting rule 1 and 2 only): ***SELECT count(*) FROM calculscd WHERE changed='$n' AND series='$series' AND (stop='0' OR stop='2')***
- The total number of free CDs of systems that have a ΔE better than a certain value ($v, in kJ/mol): ***SELECT COUNT(DISTINCT binarymin) FROM calculscd WHERE deltaE<'$v' AND series='$series'***
- Number of untreated sub-trees: ***SELECT count(*) FROM calculscd WHERE series='$series' AND stop='0'***
- Total number of treated sub-trees: ***SELECT count(*) FROM calculscd WHERE series='$series' AND (stop='2' OR stop='3')***
- Recovery of the generation of a certain experiment '$ID': ***SELECT generation FROM calculscd WHERE ID='$ID' LIMIT 1***
- Recovery of its ΔE: ***SELECT deltaE FROM calculscd WHERE ID='$ID' LIMIT 1***
- Recovery of the number of systems that are allowed to continue for the next generation for a certain generation Gn (respecting rule 1 and 2 only): ***SELECT count(*) FROM calculscd WHERE generation='$Gn' AND series='$series' AND (stop='0' OR stop='2')***
- Recovery of the number of systems of a certain generation Gn: ***SELECT count(*) FROM calculscd WHERE generation='$Gn' AND series='$series'***
- To find the average positive variation of ΔE (ΔΔE): ***SELECT AVG(deltadelta) FROM calculscd WHERE series='$series' AND deltadelta>0***
- To find the average negative variation of ΔE (ΔΔE): ***SELECT AVG(deltadelta) FROM calculscd WHERE series='$series' AND deltadelta<0***

- With phpMyAdmin, ordering the experiments of series ‘$series’ by decreasing stability (i.e. starting from the lowest ΔE): ***SELECT ID,deltaE,stop FROM `calculscd` WHERE series='$series' ORDER BY deltaE.*** The change of the ‘stop’ field value (with phpMyAdmin, by double-clicking on it) to 3, unlocks the bypass of rule 2 for its possible (allowed by rule 3) descendants. Then, typing the value of the ‘ID’ field into a form in the web page generated by the PHP software permits one to directly access to this experiment (without having to navigate into the tree, an even if not allowed by rule 1) to calculate its descendants (if not treated yet, and even if not allowed by rule 2).
- To calculate the average ΔE of a certain series: ***SELECT AVG(deltaE) FROM calculscd WHERE series='$series'***
- To calculate the best ΔE of 1-CD systems (used as the limit to determinate the 2-CD systems that are better than any 1-CD system): ***SELECT MIN(deltaE) FROM calculscd***
- Then, to calculate the number of 2-CD systems that are better than any 1-CD system: ***SELECT COUNT(*) FROM calculs2cd WHERE deltaE<(SELECT MIN(deltaE) FROM calculscd)***

Database table design

The tables, which are attached to a database must contain all the identifying values of an experiment (one row represents an experiment). The columns represent the state information, the values of certain calculations, but also the links between the experiments which derive from one another and form a tree, this one consisting of parent-descendant couples attached to each other. Table S3 is part of the database table "calculscd" which contains information for 9767 1-CD system experiments. The 8 (+2) series / trees are stored in this table, the "series" field allowing one to select only the experiments concerning the desired series (tree).

- The 'ID' field represents the unique experiment number (key), assigned by the database management system when creating a new experience row in the table (the 'AUTO_INCREMENT' attribute added to the table after its creation by an SQL command 'ALTER TABLE' allows this uniqueness, see above).

- The "Parent ID" field indicates the parent experiment (its "ID" field) from which the current experiment is derived by modifying the methylation of one of its 21 oxygen atoms. It allows navigation in the tree going back to the previous generation (Gn→Gn-1) thanks to the PHP

program and the hypertext links displayed in the web page it creates. A zero value indicates that this is the initial CD G0 (no parent).

- Conversely, searching for the value of the 'ID' field of a parent experience in the 'Parent ID' field of all the rows in the table allows you to find its descendants (downward navigation Gn→Gn + 1).

- The modified oxygen atom in the offspring is indicated in the 'changed' field, a positive value indicating methylation, and a negative value indicating demethylation.

- This change is reflected in the state attributes 'O1' through 'O21' which represent the methylation state of the corresponding oxygen atom. The values taken are: 0 = initially unmethylated and unmodified, 1 = initially methylated and unmodified, 2 = changed once to demethylated, 3 = changed once to methylated, 6 = unmethylated and 7 = methylated. Values 6 and 7 represent the last change when the possibility to change several times the methylation state of certain oxygen atoms was allowed and done by bypass of rule 2). For this reason, a unicity constraint in the table (unicity index set on O1… O21 + series) is useless, because inoperative to control respect of rule 3 (done by the PHP program, not by the database engine).

- The 'deltaE' attribute represents ΔE and 'deltadelta' its variation between itself and its parent (ΔΔE). A calculated positive value indicates destabilization which sets the "stop" attribute to 1, indicating to the PHP program that the tree should not continue in this route / branch (rule 1). A negative value indicates a system improvement and "stop" is set to 0 to allow the PHP program to display a hyperlink in the web page to be able to calculate the descendants of the descendant. When at least one descendant has been created in the database, "stop" changes to 2 to indicate that this system is a parent. A value then changed to 3 for "stop" indicates that although we should not calculate some of its descendants (mutation already made in this route), we still allow it (bypass of rule 2).

- The 'binarymin' attribute corresponds to a very simple representation of a CD, a binary representation (but seen as a number in base 10, see algorithm below): the methylation of an oxygen atom corresponds to a value of 1 and a non-methylation (hydroxyl group) to 0. All these values represent the bits of the binary number. As a βCD is composed of 7 anhydroglucose units connected in a cycle, depending on the unit from which we start to count (but always from position 2 (glucose numbering system) of each unit and in the direction 2→3→6→2→3→6→…) we can calculate 7 binary numbers. The one selected is the smallest. Two experiments that have the same 'binarymin' attribute have in common the same CD that is placed differently around SuccFerr,

which makes it easier to search by SQL query than using the 21 fields ‘O1’ through ‘O21’. A description of binarymin and how to calculate this number (algorithm and Figure S3) can be found below.

- 'mainseries' indicates the type of insertion (moieties of SuccFerr involved + insertion side of the CD) as 'series', but 'mainseries' permits any starting CD as G0 (i.e., permits to create several series 'series' with the same insertion type but with a different starting CD), where the starting CD was fixed with 'series' and is unique. Originally, S8, S9 and S10 were sub-series of 'mainseries'=8 (but disassociated because of denaturation of the model during modelling). Now 'mainseries'='series' for all the models (see below).
- The last attribute, not shown in Table S3 for clarity, is "path". It is a string of characters which indicates, in an understandable way for a person, the chain of mutations that has taken place since G0. For example, for experiment (ID) # 7011, 'path' = " 1CD succinimide into wide side of 3-6-diMe-CD: - O ₂₀ + O ₁", we see what the G0 system corresponds to and that it has then undergone two mutations: demethylation to O_{20} (sign -) followed by methylation to O_1 (sign +). This representation makes it possible to see immediately the route followed (without having to reconstruct it with each "changed" field encountered starting from G0). It is displayed in the web page for each experiment, so that the formatting HTML tags are interpreted, and that "- O ₂₀ + O ₁" is displayed simply and cleanly "- O_{20} + O_1".

The table 'calculs2cd' (2-CD systems) follows the same model but 'binarymin' is replaced by 'binarymin1' and 'binarymin2' (for each of the two CDs) and fields O31 to O51 are added to represent the 21 atoms of oxygen from the second CD. The numbering starts at 31 because it suffices to subtract 30 to find the numbering of the first CD and to guess the numbering position of the glucose.

Table S3. Partial representation (a few rows selected) of the 'calculscd' table (1-CD systems) concerning experiments in series S8. Values in kJ/mol for deltaE (ΔE of model ID) and deltadelta (ΔΔE: ΔE of model ID - ΔE of parent model IDparent). Other fields (integers) do not have units.

ID	O1	O2	…	O19	O20	O21	changed	IDparent	deltaE	stop	series	binarymin	generation	deltadelta
6990	0	1	…	0	1	1	0	0	-110.51	2	8	1797558	0	0
…	…	…	…	…	…	…	…	…	…	…	…	…	…	…
7010	0	1	…	0	2	1	-20	6990	-249.138	2	8	1273270	1	-138.628
…	…	…	…	…	…	…	…	…	…	…	…	…	…	…
7029	0	1	…	0	2	1	-18	7010	-266.011	2	8	748980	2	-16.873
…	…	…	…	…	…	…	…	…	…	…	…	…	…	…
7039	0	1	…	0	2	1	-5	7029	-279.769	2	8	748852	3	-13.758
8882	0	1	…	0	2	1	-6	7029	-259.584	1	8	738742	3	6.427
…	…	…	…	…	…	…	…	…	…	…	…	…	…	…

For clarity, the fields 'O3' to 'O18' as well as the field 'path' are not shown. The beginning of the tree is visible: experiment 6990 corresponds to the starting wdMβCD (G0), experiments 7010 (G1), 7029 (G2), 7039 (G3) are the descendants of the main branch (G0→- 20→-18→-5→…) which continues in G4 (stop = 2). Experiment 8882 is the end (stop = 1) of a secondary route because ΔE (deltaE) has increased (ΔΔE (deltadelta)> 0).

The PHP program and the webpages

The role of the program written in PHP is to generate the web page describing part of the tree (a Gn generation experiment and all of its Gn + 1 descendants) by fetching the information to display in the database. It also manages user interactions with the page (click on a hypertext link or validate forms), calculates ΔE and updates the information in the database. SQL queries are created by the program from the data it owns and are represented as strings that are sent to the database manager by the PHP function "mysqli_query()". For the queries, the database sends back to the program a response in the form of a table which must then be traversed line by line (if several lines) by extracting the attributes (one or more columns) requested by the “SELECT” clause. For modifications to the table, the SQL commands "INSERT" (insertion of a new experiment/row) and "UPDATE" (update of one or more columns of an existing experiment/row) save the program data to the database.

The program is responsible for displaying in the web page the characteristics of an experiment (identification number ID and generation Gn) chosen by the user and, in a table, all its authorized

direct descendants. These are the Gn + 1 respecting rule 2 or authorized by bypass of rule 2, or already created (in the case of the bypass of rule 1, which is then no longer controlled for this descendant), otherwise they are not displayed. The program displays their status and possible action for them. In the case of a descendant authorized but not created, the program displays a form button to create it (will trigger an SQL "INSERT" command in the database with automatic attribution of a unique "ID" experiment number serving key). Then you have to use the Modelling software to build the model according to the instructions on the web page (parent serving as the starting model and its oxygen atom to be modified). The energies of formation of the supramolecular assembly and its constituents are then calculated, then the web form displayed for the experiments awaiting results is completed. The program receives the information from the form sent, calculates ΔE for this descendant Gn + 1 and updates the fields 'deltaE' (ΔE), 'deltadelta' (ΔΔE: variation of ΔE) and 'stop' (authorization to continue or not). The page then displays the calculated ΔE and the possible actions on this descendant according to its ‘stop’ value:

- 'stop' = 0: display a hypertext link (because authorized by rule 1) to display the detail of this Gn + 1 descendant in a web page (which replaces the current web page), which also allows one to display and calculate all its descendants of generation Gn + 2 (those allowed by rule 2).

- ‘stop’ = 1: indicates that ΔE has increased for this Gn + 1 experiment so rule 1 prohibits calculating the Gn + 2 descendants of this Gn + 1 experiment. No hypertext link is displayed to the web page describing the details of the Gn + 1 experience and therefore its Gn + 2 descendants are inaccessible and therefore not calculable (end of the route).

Bypass of rule 1:

However, it is still possible to bypass the absence of a hyperlink to the description of a Gn + 1 descendant and to calculate the Gn + 2 descendants (bypassing rule 1 in a roundabout way). A form on the web page allows you to enter an experience number "ID" to go directly to its description page without any restriction. Then, the creation of a Gn + 2 experience will automatically perpetuate this link, even if rule 1 does not allow it.

Bypass of rule 2:

By default, the web page describing a Gn experiment, only displays its Gn + 1 descendants allowed by rule 2 (oxygen atoms not having already been modified in this route). To also display those which do not respect rule 2 (bypass of rule 2), you must set the 'stop' field of the parent experiment Gn to 3 (this mode only concerns the current experiment Gn and have an influence only on the display of its descendants Gn + 1). This can be done by clicking on a hyperlink at the bottom of the web page (or by changing this value with phpMyAdmin, see above).

Rule 3 being essential, the bypass is not possible, but the future descendants which do not respect it (already calculated by a parallel branch of the tree) are displayed with the other authorized descendants and with their ΔE for information. No link is displayed to go to their description, in order to keep a spanning tree: no cycle in the tree and horizontal movement prohibited). To go to this experiment, one has to go backward in the previous generations, up to a common ancestor, then has to go forward in the branch containing this experiment (it is also possible to simply type the experiment ID into the form for direct access).

Installation of XAMPP, of the database and of the PHP software

The code of the PHP programs is accessible for a better understanding. It was deposited into the data repository (https://doi.org/10.57745/CBUPP3) and on Software Heritage passing through Hal (https://hal.science/hal-03991394).

This software (consisting of several PHP files, one for 1-CD systems (cdmodele.php), one for 2-CD systems (cdmodele2cd.php), one “include file” for functions common to both previous files (functions.inc), and CDxCD.php to calculate the best combination of two CDs to use as G0 model for series 2-CD S4). The database is needed to access and navigate into the trees, and to see examples that are cited inside the paper as ‘ID …’. An alternative method, if you just want to consult the data, is to use the static web pages (see below). After installation of the XAMPP pack, downloadable for free at URL https://www.apachefriends.org (version 8.2.0 was used in this work), download the pascal.sql file (https://doi.org/10.57745/38MYXM). Start the XAMPP control panel (if error message, launch it with administrator rights) then start ‘Apache’ and ‘MySQL’ with their “start” buttons (allow them with the firewall) and click “Admin” button for MySQL to launch phpMyAdmin (or use url http://localhost/phpmyadmin/). To install the “pascal” database, click on “home” icon to be sure to not be into another database, click “import” in the

menu, select the "pascal.sql" file on your disk, then click "Import". The imported database "pascal" appears in the left panel.

Access the PHP software typing, into a browser, the url http://localhost/cdmodele.php for 1-CD systems, http://localhost/cdmodele2cd.php for 2-CD systems (http://localhost/CDxCD.php?action=show for the special method to find the best G0 for 2-CD S4 series). This software can be freely used and modified for academic research, but in case of publication please cite the present article. For help to adapt this software to another scientific problem, the author (P.P.) can be contacted.

Useful algorithms:

Notes of main text. Calculation of the average methylation-per-glucose-unit, methylation-per-glucose-unit domains (+ extra information: percentage of Me occupancy on the narrow sides and on the wide sides of the CDs), for all systems of each series that are better than their G0 (improved systems) and their corresponding free CDs (CDs without inclusion):

The PHP code is displayed in black, the comments in green and the SQL queries in blue.

```
for(i=1 ; i≤8 ; i++) //for each series 1-CD
    nMeseries=0; //overall number of Me for this series i (systems better than G0)
    deltaELevel0 ← "SELECT deltaE FROM calculscd WHERE series='$i' AND IDparent='0'
    LIMIT 1" //deltaE of G0 system (limit to determinate improved systems)
    ntotal ← "SELECT COUNT(*) FROM calculscd WHERE series='$i'" //overall number of
    systems for this series i
    nbestg0 ← "SELECT COUNT(*) FROM calculscd WHERE series='$i' AND
    deltaE<$deltaELevel0" //number of systems better than G0 for this series i
    nMemin=21 //number of minimum Me/CD for this series=maximum possible for now
    nMemax=0; //number of maximum Me/CD for this series=minimum possible for now
    nMenarrow=0; //number of Me found on narrow side
    nMewide=0; //number of Me found on wide side
    for(j = 1; j ≤ All structures of series i that are better than their G0 system*; j++)
        nMe=0 //number of Me for this CD = 0 for now
```

```
for(k = 1 ; k ≤ 21 ; k++) //all O atoms Ok of the CD (1-CD system = 21)
        if( parity of Ok is odd ) //1, 3 or 7 => Ok is methylated
                nMe++ //one more Me found for this CD
                if( k %3 ==0 ) //numbers on narrow side: 3, 6, 9, 12, 15, 18, 21
                        nMenarrow++ //one more Me found on narrow side
                else
                        nMewide++ //one more Me found on wide side
if(nMe>nMemax) nMemax=nMe; //max number of Me/CD for this series
if(nMe<nMemin) nMemin=nMe; //min number of Me/CD for this series
nMeseries+=nMe; //total Me for this series
rateMe =nMeseries / 7 / nbestg0 //average rate of Me/anhydroglucose unit for the best systems of this series
nfreeCD ← "SELECT COUNT(DISTINCT binarymin) FROM calculscd WHERE deltaE<'$deltaELevel0' AND series='$i'" //number of distinct free CDs**
Displays calculated/found information
```

* All structures of series i that are better than their G0 system: "SELECT * FROM calculscd WHERE deltaE<'(SELECT deltaE FROM calculscd WHERE series='$i' AND generation='0' LIMIT 1) AND series='$i'"

**Free CDs: In a series, certain systems can correspond to the same CD being associated to the same moiety but with different placements (rotation around the moiety). To easily find the number of distinct CDs in the series, the bynarymin value is used as an identifier for each CD.

Note: for the SQL queries (in blue), the PHP variables as $i will be replaced by their values (1 to 8) before being sent to the database management system.

For 2-CD systems, we first must retrieve the list of union of CD1s and CD2s (CDs used as both CD1 and CD2 are counted as only one): ***SELECT DISTINCT binarymin1 FROM calculs2cd where series='$series' GROUP BY binarymin1 UNION SELECT DISTINCT binarymin2 FROM calculs2cd where series='$series' GROUP BY binarymin2***. The number of lines founds by the SQL request is the researched number of free CDs.

PHP code to retrieve the data to fill Figure 4:

Code to be copied into the PHP file (cdmodele.php) for 1CD models (not included in the published version). 2CD models works the same (PHP file cdmodele2cd.php), but replace table “calculscd” by table “calculs2cd”, change the values in array $tablebests and limit to 4 values, replace 9 by 5 in the two for loops).

```
$tablebests[1]=1553; //list of the best models for each of the 8 series
$tablebests[2]=3738;
$tablebests[3]=5051;
$tablebests[4]=5370;
$tablebests[5]=5732;
$tablebests[6]=5838;
$tablebests[7]=6040;
$tablebests[8]=7759;

$maxgeneration=0; //max generation to be displayed (for Figure 4 of the article)
for($laserie=1;$laserie<9;$laserie++) //for the 8 1CD series
{
   $ID2=$tablebests[$laserie]; //retrieves the current series
   do //searches from best model to the G0 model (decreasing generation)
   {
      $requete="SELECT IDparent,deltaE,generation FROM calculscd WHERE ID='$ID2'
LIMIT 1"; //for model $ID2, retrieves deltaE, the generation and its parent model (its ID)
      if($row=fctselect1X($requete,1,$id)) //for this handmade function, see reference
https://hal.science/hal-03991394 (or Software Heritage whose link is inside)
      {
         list($IDparent,$deltaE,$generation)=$row; //retrieves data from database.
         if($maxgeneration==0) $maxgeneration=$generation; //finds the higher generation to be
displayed in Fugure 4.
         $tabledeltaE[$laserie][$generation]=$deltaE; //saves into array for latter.
      }
```

```
        else { print("<p>ID $ID2 not found !</p>"); exit(); } //should not be.
        $ID2=$IDparent; //Continues with the parent to find its own parent.
    }
    while($generation>0); //loop until G0 is reached.
}
for($g=0;$g<=$maxgeneration;$g++) //print data ordered by generation.
{
  print("<p>G$g"); //(print the generation: G0, G1, …)
  for($laserie=1;$laserie<9;$laserie++) //then by series
    {
        if(isset($tabledeltaE[$laserie][$g])) $d=$tabledeltaE[$laserie][$g]; else $d=0; //if no value
for deltaE, replaces by 0 (0 will be erased in Excel afterwards). Otherwise, copies the value.
        print(";$d"); //writes the value.
    }
    print("</p>"); //closes the paragraph.
}
```

When the webpage finishes to display the table, copy/past it into a text file, open Excel and insert the content of the file, erase all 0 values, do the graphic.

Algorithm to calculate binarymin:

As stated above binarymin is a binary representation of the CD. Indeed, each oxygen atom (Oi with i ranging from 1 to 21 in 1-CD + 31 to 51 systems for 2-CD) can only take a binary state (methylated or not), like the bits which represent the smallest information of a computer (1 or 0). In addition, the oxygen atoms are arranged in a certain order (their numbers), as an integer encoded in the memory of a computer consists of a series of numbered bits (0, 1, 2, ...), each bit having a weight equal to 2^{number} (2^0, 2^1, 2^2, …). The value of the integer, converted to base 10, is the sum of each bit multiplied by its weight (so only bits with 1 count in this sum). By analogy, we therefore converted the configuration of a CD into a binary number. The reason for creating binarymin is to simplify the SQL query to find a particular CD. Indeed, an Oi can take 6 possible values (0, 1, 2, 3, 6 and 7), which makes the request complicated by considering the 21 Oi while the search on binarymin is simple and very fast (theoretically more again if we placed an index

on this field). This binarymin number is calculated only once, when creating the experiment in the database (inserted in the SQL query "INSERT INTO"). The binarymin2 field of the second CD of 2-CD systems is calculated similarly.

Depending on the anhydroglucose unit from which we start to count, and always starting from a position 2 (in the glucose numbering system, then 3, 6, then the following anhydroglucose unit) we can obtain 7 binarymin numbers different. We only keep the smallest. The algorithms of the two functions performing this calculation are shown below followed by the explanatory diagram.

```
//The first function converts the configuration of the CD to a binary number depending on the
starting anhydroglucose unit (Shift). Array o contains the configuration (methylation state of all O
atoms 1 to 21).
function ConvertCDtoBinaryNonUnique(o, shift)
    binary=0 //binary number to calculate
    multi=1 // → weight of the bit = 2^0 (=1 for starting bit 0)
    for(i=0 ; i<21 ; i++) //for each oxygen atom Oi (i.e., o[i])
        binary += multi * o[ ( ( 3 * shift + i) %21 ) +1 ] //adds value of the bit x its weight
        multi *= 2; //then the weight become 2^1, 2^2, 2^3, … for next bit
    return binary //send calculated binary

//The second function converts the configuration to a unique binary number by calling seven times
function ConvertCDtoBinaryNonUnique(), each one starting from a different anhydroglucose unit
(the smaller binary number on the 7 possibilities, will be saved in database afterwards). Array o
contains the configuration (methylation state of all O atoms 1 to 21).
function ConvertCDtoBinaryUnique(o)
    BinaryMin = 2097152; // 2^21 = max+1
    for(i =1 ; i<22 ; i++) o[i]=o[i] & 1; //0, 2 or 6 →0, 1, 3 or 7 →1 (conversion to 0 or 1 only)
    for(i=0 ; i<7 ; i++) //for each starting anhydroglucose unit
        binary=ConvertCDtoBinaryNonUnique(o, i) //start counting from anhydroglucose
        unit i
        if(binary < BinaryMin) BinaryMin=binary //retains the smaller one
    return BinaryMin //and send it
```

For example, the best 1-CD series 8 experience (ID = 7759) has an official binarymin of 9346, which corresponds, following the order of the numbers, and starting at number 1, to an H-Me-H-H-H-H-H-Me-H-H-Me-H-H-Me-H-H-H-H-H-H-H string starting the count at O1. By converting the string into 0 (H) and 1 (Me), and by returning the string, to write the binary number in conventional notation (decreasing weight), we obtain 000000010010000010, that is to say 9346 in base 10 notation. The other combinations starting the count at O4, O7, O10, O13, O16, O19 (the other positions numbered 2 in glucose notation), give larger numbers, so 9346 is retained.

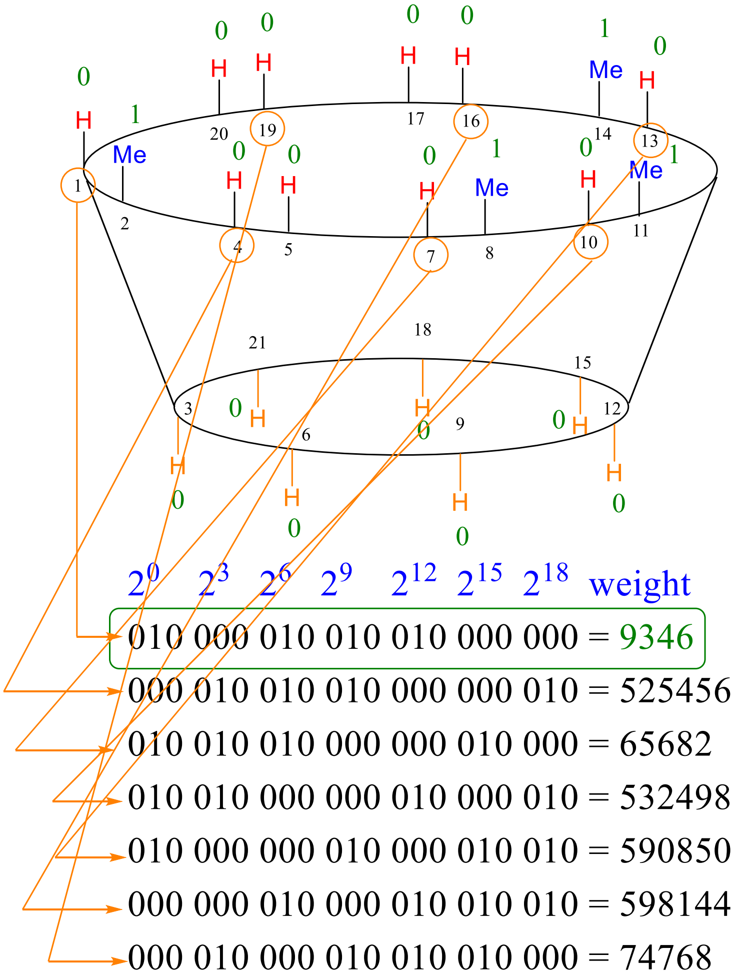

Figure S3. Determination of the binarymin code, the minimum of the 7 binary numbers calculated when starting from position 2 (glucose numbering system) of each of the 7 anhydroglucose units (orange circles). Binary numbers are represented from left to right to be consistent with the way of reading the CD but should be reversed in conventional binary notation.

With this binarymin number, it is easy to find the number of free CDs of a series ‘s’ (when freed from supramolecular assembly, i.e., the number of distinct CDs):

- For 1-CD series (remark: DISTINCT eliminates doubloons): *SELECT COUNT(DISTINCT binarymin) FROM calculscd WHERE series=’s’*.
- For 2-CD series: 1) First, we recover the list of total CDs by the intersection (UNION, that also eliminates doubloons) of CD1 and CD2: *SELECT DISTINCT binarymin1 FROM calculs2cd WHERE series=’s’ UNION SELECT DISTINCT binarymin2 FROM calculs2cd WHERE series=’s’*, 2) then we look at the number of results the database displays.

To collect all the free CDs of the 12 series (1-CD and 2-CD) to determine in how many series they are involved, it is necessary to make the intersection of the free CDs of the 1-CD and those of 2-CD systems (by making the intersection of CD1 and CD2), by the SQL command:

SELECT DISTINCT binarymin FROM calculscd GROUP BY binarymin UNION SELECT DISTINCT binarymin1 FROM calculs2cd GROUP BY binarymin1 UNION SELECT DISTINCT binarymin2 FROM calculs2cd GROUP BY binarymin2.

Then, number of series a CD is implicated into (replace b by the researched binary value, and this for all the free CDs found above): ***SELECT COUNT(DISTINCT series) FROM calculscd WHERE binarymin='b'*** (for 1-CD) or ***SELECT COUNT(DISTINCT series) FROM calculs2cd WHERE binarymin1='b' OR binarymin2='b'*** (for 2-CD).

Insertion of a new experiment into the table ‘calculscd’ (called by the PHP software by its “handmade” fctinsert() function; the variable ‘$num’ receives the unique ‘ID’ value attributed by the database).: $num=fctinsert("***INSERT INTO calculscd VALUES ('0','$o[1]','$o[2]','$o[3]','$o[4]','$o[5]','$o[6]','$o[7]','$o[8]','$o[9]','$o[10]','$o[11]','$o[12]', '$o[13]','$o[14]','$o[15]','$o[16]','$o[17]','$o[18]','$o[19]','$o[20]','$o[21]','$signe2$oxygenet ','$path $signe O_{$oxygenet}','$IDparent','0','0','$series','$NumUniqueCD', '$generation', '0' ,'$mainseries')***",0,$id).
First parameter (ID): 0 indicates to the database to attribute a new and unique ID to the new record. Second to 22th parameters (O_1 to O_{21}): $o is the table that contains the values of all O atoms $O_{1...21}$. Thus, $o[i] represents oxygen atom number i into table $o of the PHP program.

23th parameter (changed = $signe2$oxygenet): O atom ($oxygenet is its number) that was changed with sign ($signe2) – for demethylation and sign + for methylation.
24th parameter (path): "$signe O_{$oxygenet}" is appended to the end of the string 'path' to indicate in a comprehensive way, the O atom of the parent experiment that is changed in this descendant. The HTML tags < > are interpretated when displaying path in webpage (O atom number ($oxygenet) is set to subscript).
25th parameter (IDparent'): ID of the parent experiment this experiment derivate from.
26th parameter (deltaE: ΔE): Set to 0 to indicate this value is to be calculated (pending calculation).
27th parameter (stop): No decision yet if the experiment can continue in next generation (waiting for calculation).
28th parameter (series). Series 1 to 10.
29th parameter (binarymin): Calculated and stored in PHP variable $NumUniqueCD.
30th parameter (generation): generation was incremented by the PHP program (generation of its parent + 1).
31th parameter (deltadelta: ΔΔE): 0 for now (waiting for calculation).
32th parameter (mainseries): Permits to gather series that have the same inclusion type (same moiety + same inclusion side of the CD) but starting from a different CD for its G0 model. This is the field used (with O1 – O21) to search for an existing model to avoid creating a doubloon into the table of the database (rule 3). For details on its utility, see below (1-CD S9 and S10).

The database was first opened calling the PHP function mysqli_connect() (into include file functions.inc that was used by the three PHP files) that returns a value (stored by the PHP program into variable '$id', if the database was opened successfully) that serves to link PHP and the database it has opened. This variable $id should be furnished with each query sent to the database. It should be noted that this variable $id should not be confused with variable $ID (variables are case-sensitive) that represent the number of a model (its ID field inside the database table).

All queries are sent by the PHP program to the database management system using the native "$res=mysqli_query($id,$requete);" command, where: - $id is the handler for the database (see just above), - $requete is a text variable containing the SQL query as a text. The result of the query is returned and stored into a variable (here $res), whose value is 'false' if the query failed (SQL syntax error for example).

For illustration: The handmade PHP function fctinsert(…), is used to send a 'INSERT INTO' SQL request to the database to save the new pending experiment by creating a new row (see above). This function uses the "fctsql($requete,$stop,$id);" command (where fctsql is also an handmade function, where $requete and $id were described above and $stop indicates if the PHP program should stop or not if the query failed). This last function calls the native mysqli_query() PHP function.

Example of update of several attributes of the experiment '$ID' in the table of the database:
When the calculation of the pending experiment was done by Spartan, and the energies were entered the form in the webpage, the PHP program receive the data, calculates deltaE
(ΔE calculated and stored in PHP variable $e), and allows or not to continue in next generation ('stop'), depending on calculated 'deltadelta' (variation of ΔE (ΔΔE) calculated and stored in PHP variable $deltadeltacal):
UPDATE calculscd SET deltaE='$e', stop='$stop', deltadelta='$deltadeltacal' WHERE ID='$ID' LIMIT 1.
LIMIT 1 was added to the request to limit the research of the experiment in the database to only one (there is only one to be modified, so searching all the database when one was already found is a loss of time).

Case of 1-CD series 9 and 10

A question that arises using our method is: "Is this technique able to detect the best models?" As said in the article, we are not looking for the global minimum, because no complex CD will be synthesized to make this assembly. However, an evaluation of the effectiveness of the method is required to appreciate the convergence towards a local minimum close to the global minimum (which could theoretically only be found and proven by making a calculation on all the models, which is currently impossible). To help answer this question, we decided to make a modification to the method and the web application first used for 1-CD S1 to S8. Two new 1-CD series have been made (S9 and S10), but with the same inserts as for the S8 series (reminder: Succ inserted into the wide side of 3,6-diMe-CD). The goal was not only to see if we converge to a better minimum than S8 starting from a different point into the combination space, but also to find if we converge to some local minima already found by S8. Starting from different CDs (for S9, from 2-

Me-CD which is the exact inverse of 3,6-diMe-CD, and from 3-Me-CD for S10), the 2 series each formed a tree of mutations evolving in parallel, with only one exception: rule 3 (no duplicates) applies for S8 UNION S9 UNION S10 thanks to the creation of a new field in the database, "mainseries". For S8, S9 and S10, this field has been set to 8 (mainseries = series for others), indicating that the PHP program must now rely on this field, and not on the "series" field to detect duplicates before they are created. It is this rule that must make it possible to see the collision between these 3 series/trees and therefore to see if we converge well towards the same models. However, by analyzing these models, we realized that the Succ group was not well inserted into the CD, and worse, that after a few generations, it was the Fc group that was partially inserted automatically into the CD cavity (see ID 9191 for S9 and 9690 for S10), which corresponds more to S2 than to S8. The S9 and S10 series have therefore been distorted and have been disassociated from S8 in the database (they became independent, setting mainseries = series using a SQL query). They have also not been associated with S2 because some of their models are intermediate between S8 and S2 and moreover, the control of duplicates was not made with respect to S2. These two series have therefore not been included in Table 1 on main text, but their models are provided for information as for the other series in the form of XYZ files. We wanted to know more about the reason for this failure and noticed that from G0, Succ is almost not inserted into the cavity. The reason is due to hydrogen bonds between one (e.g., ID 8996) or two carbonyls (ID 8997) of the imide which anchor the succinimide group at the cavity entrance. However, methylation of one of these OH groups on the wide side of the CD, even though breaking the hydrogen bond, does not allow Succ to penetrate deep into the cavity because the carbonyl then finds another hydroxyl to replace it (for example ID 8909 – (+ O_8) $\rightarrow$ ID 8914 where O_8 is replaced by O_{12}). Forced manual insertion of Succ when modifying the model does not work around this problem; when calculating the geometry, Succ is still sticking out of the cavity. We have therefore demonstrated that this method can succeed or fail depending on the CD chosen for the G0 generation, and that it is preferable to start from the models with the lowest ΔE for G0, i.e., from the most suitable CD, as we instinctively did for the others before the S9 and S10 series. However, the method of starting from different points into the combination space, to browse larger space (but remaining reasonable), is validated, since it could permit to get around falling into a local minimum that hampers to reach a better minimum.

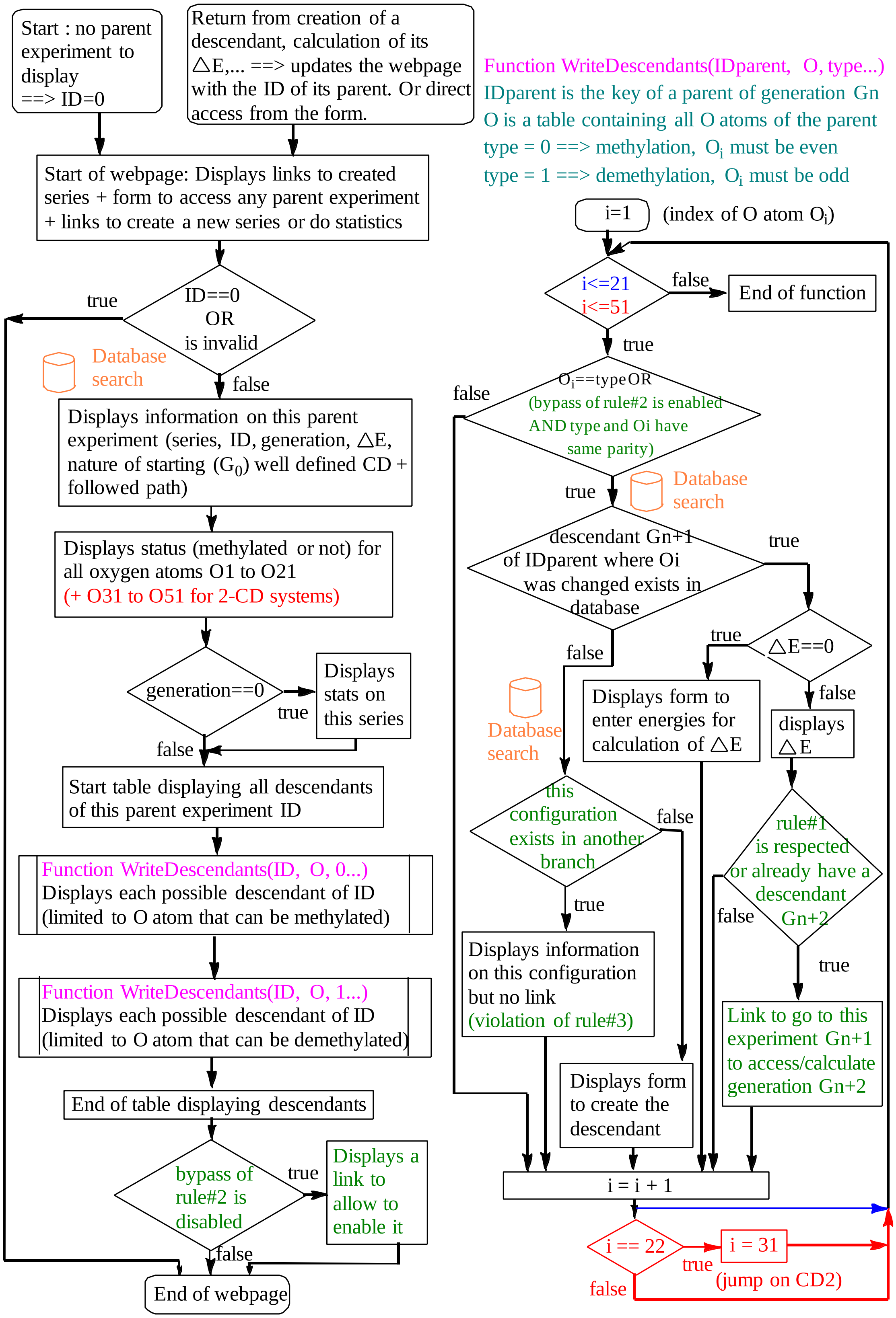

Figure S4. Algorithm of the two PHP programmes. Drawing in blue is specific to 1-CD systems and drawing in red to 2-CD systems. Effects of rules 1,2,3 and their bypasses are drawn in green.

Examples without XAMPP (static webpages)

To be able to see the results of the web application without installing XAMPP, all the corresponding static webpages were created by the C program (see below) and can be seen using a navigator. These webpages can be found into the data repository and into Software Heritage (links in the first page of this document) as a compressed archive. The archive should be downloaded, unzipped, and almost any webpage (preferably the first HTML file) can serve as an entry point to then reach any model. Indeed, the links work, permitting to navigate off-line into the models.

C program to control the models

This C program works on 1-CD series or on 2-CD series depending on a constant set in the program ("#define CD1OR2 1" to work on 1-CD series, "#define CD1OR2 2" for 2-CD series) before compiling it. It needs the corresponding database table to work. For this purpose, the tables were exported from phpMyAdmin as tab files (openable with a text editor, alternatively with Excel). In fact, these files were saved from phpMyAdmin as .csv files then renamed as .tab files (extension changed) to be compliant with the data repository system (to be visible on-line). The needed files to be opened by the C program are "calculscdprog.tab" for the "calculscd" table and "calculs2cdprog.tab" for the "calculs2cd" table. These files have tabulations as separator and the fields are not escaped by quotes. It should be noted that these tab files only differ from the "calculscd.csv" and "calculs2cd.csv" files (also in the repository) by their format (separators = comma, fields escaped by quotes), format requested by the repository for CSV files. These files make it easy to recreate the database regardless of the OS or database management system. But the C program can also read the .tab file, extracts its content, and fills a structure (typedef struct { … } table;) whose instance is called "database". Some of the SQL queries executed by the PHP program are simulated by the C program by accessing the fields of this structure (or an adapted copy where the Oi fields are simplified (0 or 1 only) to simplify the simulated SQL query).

The program also needs to have access to the folders where the models' files are saved. When a parent model was used to create its descendants in Spartan, a folder was created inside the same folder than the parent and all descendants were put inside, and so on for several generations. When the XYZ files were created from Spartan, these files were saved into the same folder as its original Spartan model file. The program can know and access to the corresponding folder and file of each model thanks to the "path" field of the table/.tab file. However, some information is lost in XYZ files, such as chemical bonds and atom labels. Nevertheless, these text files are easily openable by handmade software, which can recreate the lost chemical bonds according to the interatomic distances, and then to recreate the molecule by linking neighboring atoms and control if the files and the database match.

Although the PHP program could analyze these models, this procedure is not especially applicable because of the constraints applied to a web application, which thus shows its limits. A "standalone" program is therefore desirable, and we have carried out this audit program in the C language. The data can be easily retrieved, each line representing a model.

The C program was created for several purposes:

- As stated above, for fast consultation of the trees of models displayed into the dynamic webpages by the PHP program, but without the need to use it, static webpages, one for each model, were furnished into the repository as clones of the dynamic webpages. Saving all pages one by one from the browser would have caused several issues: - It would have taken a long time, - Manually giving to the saved webpages a name related to their ID would have caused some errors, - The original links would have been unsuitable to operate off-line, hindering the navigation into the trees, or would have necessitated to manually adapt the links for each page. To create these static webpages, the behaviour of the C program was copied on the code of the PHP program but adapting the links.
- When using a parent model to create its descendants in Spartan, obviously errors are unavoidable. The errors can be: - changing the wrong oxygen atom or forgetting to change it, - accidentally erasing an atom (this unvalidated a whole series having more than 1000 models that had to be redone because the G0 was wrong and the error has spread into descendants), - starting from the wrong parent, forgetting to convert some Spartan models into XYZ files… The C program having access to the

"path" field of the database, was able to determinate the path to the file, to look for its presence, to open it and to create a modified copy. The modifications are:

- Gathering all XYZ files into the same folder "XYZ" (the one that was compressed into an archive deposited into the repository), but each series being into its own folder "aCDSb" where "a" is the number of CDs (1 or 2) and "b" is the series.
- Changing the name of the copy to comply with the format "a-CD-Sb-ID-c.xyz" where "a" and "b" are those described above and c is the ID of the model.
- Fixing an error of Spartan that does not respect the format for XYZ files. Indeed, the two first lines are missing when saving XYZ files from Spartan 14 (not from Spartan 20). These two lines were added with the first line being the number of atoms into the model/XYZ file, and the second line being a facultative comment (that was filled with information about the model, based on its "path" field).
- Removing the two pseudo atoms "Lig" than Spartan adds to connect ligands to the metal atom, here Fe connected to two Cp (cyclopentadienyl), but that are confused with lithium by other modelling software.
- Accessory, reducing the size of the XYZ files by replacing several consecutive spaces by a unique tabulation.
- Finding the real name of the original XYZ file. Indeed, for high generation models, the path to the file became too long, provoking errors in Windows 10. The only possibility, if we want to keep the path to the folder, was to reduce the name of the original XYZ file to reduce the total path (folders path + file name). To do this, the beginning of the file was truncated. The C program needed to scan the last folder of the path to find the real name of the file finishing with "G0.xyz" for the G0 model, "+a.xyz" for models finished by a methylation or "-a.xyz" for models finished by a demethylation, where "a" is the modified oxygen atom.

Then, the program reads the new XYZ file and writes into a file (message.txt) all errors that were found. To check if the configuration of the CD(s) was correct, the "binarymin" of the XYZ file was calculated and compared to that of the database: Starting from the first found

anomeric carbon atom, the program progress from neighbour atom to the next one, checks the methylation state of the three important oxygen atoms on its way, and this for each glucose unit. It should be noted that this method does not guaranty to find all errors for the G1 models, because three groups of CDs have in common the same (free) CD (those having the inversion of methylation on the same number, in glucose numbering: 2, 3 and 6). Moreover, the number of atoms with a certain valence where also counted (H, O, C and N).

- Hydrogen bonds were found to be important for the stability of the supramolecular models, in particular hydrogen bonds implicating the iron atom of ferrocene. We used the C program to do extra statistics based on these hydrogen bonds but limited to intermolecular bonds (between the inserted molecule and a CD). To find these bonds, we fixed the limit to 2 Å (#define HBONDLIMIT2 4.0 in the C program is the square of this value, because with wanted to compare squares of distances to avoid using the square root function that is time consuming). For SuccFerr the atoms involved in these bonds are the iron atom, the two phenols (-O- and -H independently) and the two carbonyls (=O) of the imide group. For CDs these are the hydroxyls (-O- and -H independently), the ethers (-O-) and the anomeric group (-O-). The C program created files Hbonds-aCD.txt, where “a” is the number of CDs (1 or 2). Then, it has reread these files to make a webpage containing statistics on these bonds, including the found clamps (“TableofHbondsbySeries-aCD.html” where a is the same as above, inside the compressed archive where the other webpages are gathered).

Details on the C program and call to functions

The C program flows in this order:

- Program starts.
- Creates the structure “table” and creates an instance: “database”. It will contain all the fields of the MariaDB table (“calculscd” for 1-CD and “calculs2cd” for 2-CD models) plus complementary fields that are calculated on-demand by the PHP program but that will be calculated once by the C program and stored in “database”. Its size is set to 9800 (#define NLINESMAX 9800) for 1-CD or 2-CD models.
- Opens the "message.txt" file for writing. All found errors (if any) will be written inside.
- Opens "Hbonds-aCD.txt" for writing (where “a” is the number of CD). All H bonds of interest and Me (methyl groups) close to Fc (ferrocene) will be written inside.

- Calls “ReadCSVToTable()”:
    - Open the csv file ("calculscdprog.tab" or "calculs2cdprog.tab").
    - Reads each line of the file (one line = one model) and fills the database structure for each record “ID”. Since the table was ordered, ID = number of the record.
    - If constant “MAKEXYZFILES” was set to 1 (default, allows to create adapted XYZ files):
        - Calls function “CopyConvertedXYZFiles()” that reads, modifies and copies the new corresponding XYZ file for this experiment, knowing its ID and path.
        - Calls function “ChecksBinaryConfiguration()” that reads the new adapted XYZ file, calls function “VerifyCount()” that counts the number of atoms by type and valence, checks if incoherences exist into the XYZ file (writes errors in file "message.txt"), calls function “CalculationOfBinary()” that calculates the “binarymin” for the CD (then the “binarymin” for the second CD, for 2-CD models).
        - Checks for errors (if calculated “binarymin” for the XYZ file and in database are different) and if any, writes in file "message.txt".
        - If constant “MAKEHBONDSSTUDY” is set to 1, calls function “FindFe()” to search for the iron atom, calls function “FindThe2Carbonyls()” to find the two carbonyl groups (imide), calls function “FindThe2Phenols()” to find the two oxygen atoms and the two hydrogen atoms of the two phenol groups. Then calls function “FindHBonds()” for each type of hydrogen bond, that are written into file "Hbonds-1CD.txt" or "Hbonds-2CD.txt". Calls function “FindNumberOfMeCloseToFe(Fe)” to count the number of Me close to ferrocene (to Fe) and to write this information into file "Hbonds-1CD.txt" or "Hbonds-2CD.txt".
    - Determines how many series are inside the CSV file (store into variable “NumberOfSeries”) then closes it.
- Closes file "Hbonds-1CD.txt" or "Hbonds-2CD.txt".
- For each series, calls function “TreatTree()” to complete the database structure by some calculations (for values not in the original database but calculated on-demand by the PHP program):

- Determines, in “database”, the area where the models of this series are located (to increase the search speed by reducing this area).
- Calls the recursive function “TreatTreeRecursive()” that browses this model and all its descendants (sub-branch starting from this model) to determine all fields not in the original database: “best” (number of descendant models that are better than this model), “pending” (number of models that are pending, i.e., whose ΔE was not calculated yet), “finished” (all descendants are blocked by rule 1 or not, dead end or not for this sub-branch), “max_generation” (the higher generation reached inside this sub-branch), “bestdeltaE” ” (the higher ΔE reached inside this sub-branch), “descendants” (the number of descendants this model has inside the sub-branch this model is the ancestor).

- If constant MAKEHBONDSSTUDY and constant MAKEXYZFILES are set to 1, creates a stat report on H bonds by reading file “Hbonds-1CD.txt” or “Hbonds-2CD.txt”.
  - Opens file “Hbonds-1CD.txt” or “Hbonds-2CD.txt” for reading.
  - Calls function “CreateHBondsStatReport()” to create the report uniquely with data of the txt file.
    - Opens file "TableofHbondsbySeries-aCD.html" (“a” = number of CDs = 1 or 2) for the webpage containing the statistics about hydrogen bonds and Me close to Fc for each series. Starts to write the beginning of its HTML tag + information.
    - Finds each series inside the text file.
    - For each series, calls function “CalculatesLimitDeltaE()” to Find the minimum and maximum ΔE for this series and the average value (will be needed to find if a model should be classified as good or worse model).
    - For each series, reads each model, class it as good or worse models, counts the number of hydrogen bond for each type and the average number of Me close to Fc found. Writes the values into a table into the webpage, with a column for the good models and a column for the worse models.
    - Closes the HTML file.
  - Closes the text file.
- If constant MAKEHTMLFILES was set to 1 (creates the webpages for all the models)

- Calls function “AdaptedDatabase()” to create a copy of array “database” (dynamic allocation) with simplified fields Oi: 0 or 1 only. It permits to simplify the query that will imitate the SQL query to find if a model exists in database.
- Calls function “CreateTheWebPages()”.
  - Determines, in “database”, the area where the models of this series are located (to increase the search speed by reducing this area).
  - For each series, Calls function “CreateOneWebPage()” that creates the webpage for the model whose ID is furnished in parameter. It opens the webpage, includes into the webpage the beginning that is common to all webpages, then the content that depends on the database. It calls function “WriteDescendants()” for the descendants of this model that were methylated, then for the descendants that were demethylated, then closes the webpage.
- Free the memory allocated for the copy of “database”.

- Closes text file “message.txt”.
- End of program

Case of the best models of 1CD series 8 and series 2 (figures S5 and S6)

Comparison of best model of 1CD series 8 calculated in PM3 (Spartan 14, Figure S5a) and in DFT (Spartan 20, Figure S5b):

Note: This DFT calculation (Figure S5b, ωB97X-D, 6-31G*, medium=water, option PCMRAD=FE~2.2, starting from the PM3 model) is different from Figure 4 (main text) that was calculated with the 6-311G* basis set and Gaussian 09, but gave comparable results.

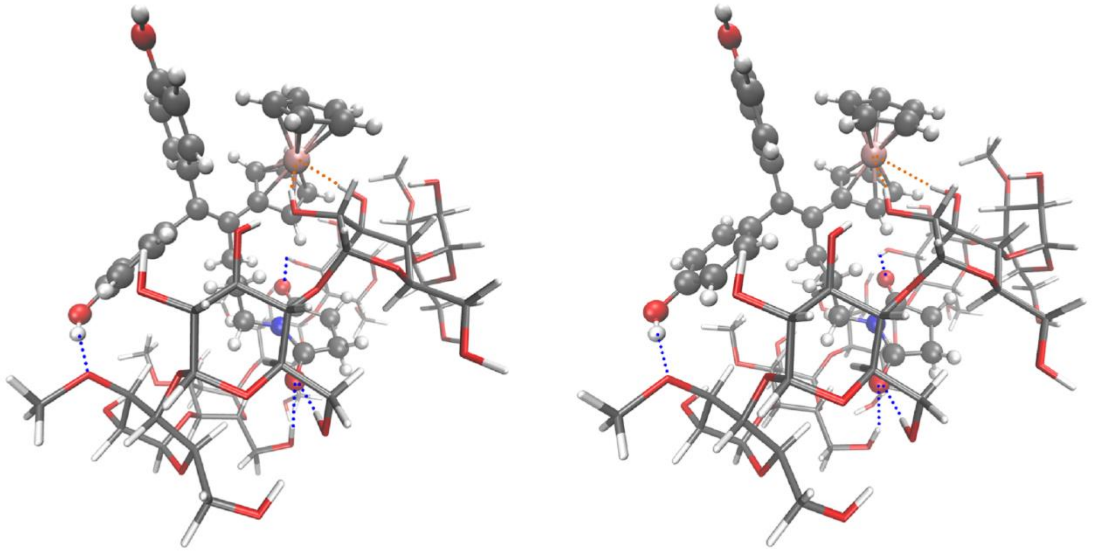

(**a**) (**b**)

Figure S5. Molecular models of the best 1-CD system (series S8, ID 7759, Succ entering by the wide side of the CD). SuccFerr is represented with a "ball-and-stick" model and the CD is represented with a "licorice" model. Orange dashed lines (clamp between the iron atom and O_{19}–H and O_{20}–H) represent the two atypical hydrogen bonds between the iron atom and the CD. Another clamp between one of the C=O group and O_{12}–H and O_{18}–H is also visible, as bonds between the phenol group (Ph2) and O_{14}–Me and between the second C=O of imide and O_5-H (blue dashed lines). The numbering is not shown. (**a**) Computed with Spartan 14 at the semiempirical PM3 level of theory (i.e., in vacuum), with length of Fe-H bonds ≈ 1.9 Å and angles of O_{19}-H…Fe and of O_{20}-H…Fe ≈ 167° and 177°. (**b**) Computed with Spartan 20 at the DFT level of theory, with length of Fe-H bonds ≈ 2.6 Å and angles of O_{19}-H…Fe and of O_{20}-H…Fe ≈ 165° and 169°.

Best models of 1CD series 2 (PM3) (this calculation did not succeed in DFT):

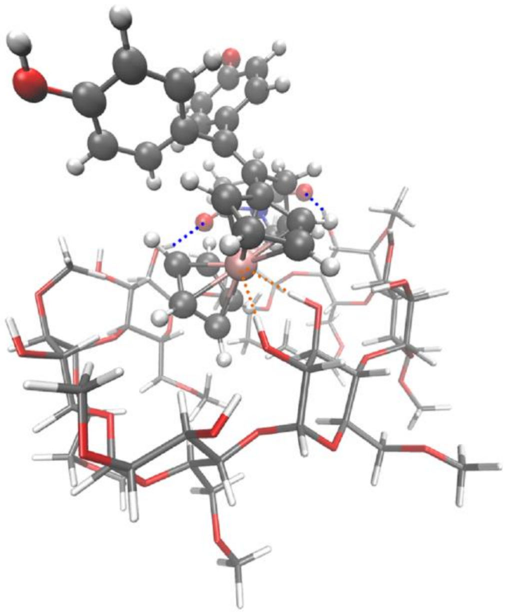

Figure S6. Molecular model of the best system of 1-CD series S2 (ID 3738, Fc included by wide side of the CD, ΔE = -261 kJ/mol) computed at the semiempirical PM3 level of theory. SuccFerr is represented with a "ball-and-stick" model and the CD is represented with a "licorice" model. The 2 hydrogen bonds between the iron atom and the CD are represented by orange dashed lines (clamp between the iron atom and O_{10}–H and O_{11}–H, length ≈ 1.9-2.0 Å). Hydrogen bonds also exist between each C=O of imide group and -O_1-H and -O_{17}-H (blue dashed lines).

Table S4. Second-order perturbation stabilization energies for the NBOs involving atoms taking part in the atypical H-bond Fe-HO interactions. NBO labels: LP = Lone pair, LP* = Antibonding lone pair, CR = Core.

**Fe ----- H_{210} – O_{19}**	**NBO Donor**	**NBO Acceptor**	**$E^{(2)}$ (kj/mol)**
	LP (1) (Fe)	Rydberg (1) (H_{210})	0.92
	LP (1) (Fe)	Rydberg (4) (H_{210})	0.21
	LP (1) (Fe)	σ* (1) (H_{210} – O_{19})	1.80
	LP (2) (Fe)	σ* (1) (H_{210} – O_{19})	1.67
	LP (3) (Fe)	σ* (1) (H_{210} – O_{19})	0.33
	LP* (6) (Fe)	Rydberg (1) (H_{210})	25.48
	LP* (6) (Fe)	Rydberg (2) (H_{210})	2.80
	LP* (6) (Fe)	Rydberg (3) (H_{210})	6.69
	LP* (6) (Fe)	Rydberg (4) (H_{210})	14.85
	LP* (6) (Fe)	Rydberg (5) (H_{210})	0.75
	LP* (7) (Fe)	Rydberg (2) (H_{210})	0.67
	LP* (7) (Fe)	Rydberg (4) (H_{210})	0.38
	LP* (8) (Fe)	Rydberg (1) (H_{210})	1.97
	LP* (8) (Fe)	Rydberg (3) (H_{210})	0.29
	LP* (8) (Fe)	Rydberg (4) (H_{210})	1.38
	LP* (9) (Fe)	Rydberg (5) (H_{210})	1.51
	LP* (6) (Fe)	Rydberg (1) (O_{19})	0.50
	LP* (6) (Fe)	Rydberg (3) (O_{19})	1.80
	LP* (6) (Fe)	Rydberg (4) (O_{19})	1.88
	LP* (6) (Fe)	Rydberg (7) (O_{19})	0.29
	LP* (7) (Fe)	Rydberg (1) (O_{19})	2.09

LP* (7) (Fe)	Rydberg (4) (O_{119})	1.71
LP* (8) (Fe)	Rydberg (3) (O_{19})	0.21
LP* (9) (Fe)	Rydberg (7) (O_{19})	0.75
LP* (8) (Fe)	σ* (1) (H_{210} – O_{19})	4.48
σ (1) (H_{210} – O_{19})	LP* (6) (Fe)	7.49
σ (1) (H_{210} – O_{19})	LP* (7) (Fe)	0.96
σ (1) (H_{210} – O_{19})	LP* (8) (Fe)	1.17
σ (1) (H_{210} – O_{19})	LP* (9) (Fe)	0.21
σ (1) (H_{210} – O_{19})	Rydberg (9) (Fe)	0.29
LP (1) (O_{19})	LP* (7) (Fe)	0.25
LP (2) (O_{19})	LP* (6) (Fe)	0.67

Fe ----- H_{193} – O_{20}

CR (1) (Fe)	Rydberg (4) (H_{193})	0.21
LP (1) (Fe)	Rydberg (1) (H_{193})	1.34
LP (1) (Fe)	Rydberg (3) (H_{193})	0.63
LP (1) (Fe)	Rydberg (4) (H_{193})	0.92
LP (3) (Fe)	Rydberg (3) (H_{193})	0.25
LP (1) (Fe)	σ* (1) (H_{193} – O_{20})	1.30
LP (3) (Fe)	σ* (1) (H_{193} – O_{20})	1.60
LP* (6) (Fe)	Rydberg (1) (H_{193})	23.68
LP* (6) (Fe)	Rydberg (3) (H_{193})	18.33
LP* (6) (Fe)	Rydberg (4) (H_{193})	38.99

LP* (6) (Fe)	Rydberg (5) (H_{193})	0.42
LP* (7) (Fe)	Rydberg (2) (H_{193})	1.63
LP* (8) (Fe)	Rydberg (1) (H_{193})	0.79
LP* (8) (Fe)	Rydberg (3) (H_{193})	0.84
LP* (8) (Fe)	Rydberg (4) (H_{193})	0.96
LP* (9) (Fe)	Rydberg (5) (H_{193})	12.76
LP* (8) (Fe)	σ* (1) (H_{193} – O_{20})	4.43
σ (1) (H_{193} – O_{20})	LP* (6) (Fe)	6.23
σ (1) (H_{193} – O_{20})	LP* (8) (Fe)	2.38
σ (1) (H_{193} – O_{20})	Rydberg (1) (Fe)	0.25
σ (1) (H_{193} – O_{20})	Rydberg (11) (Fe)	0.25
LP (1) (O_{20})	LP* (7) (Fe)	0.21
LP (1) (O_{20})	LP* (8) (Fe)	0.21
LP (2) (O_{20})	LP* (6) (Fe)	0.63

Description of the trees of modelling and of the web application to manage them

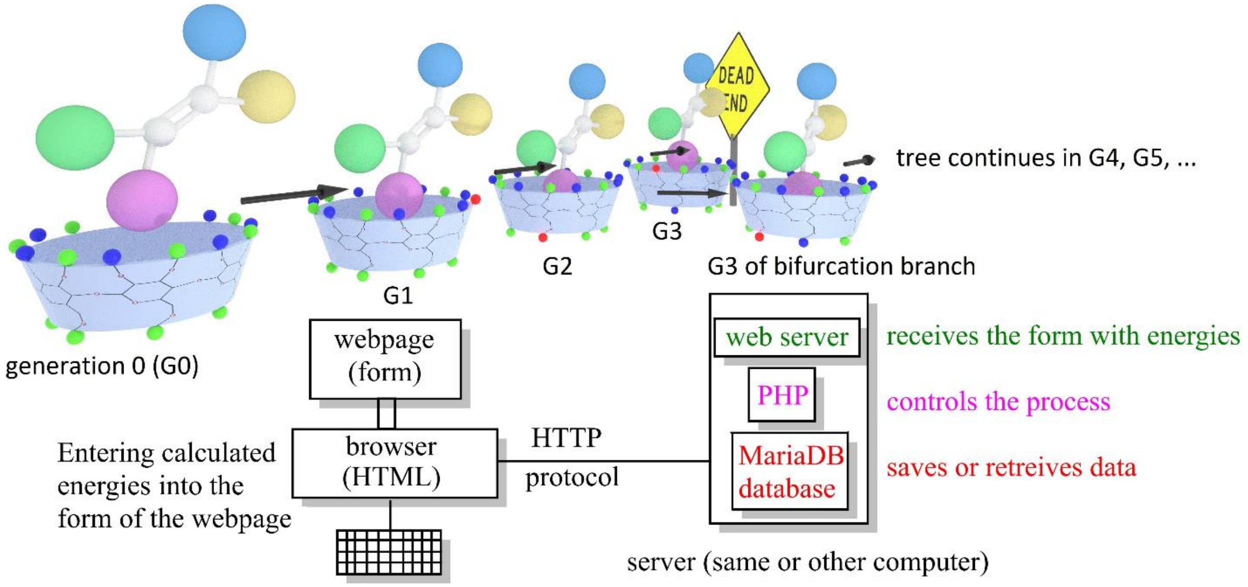

Explanation: In a very schematic view, the molecule (SuccFerr, with its central double bond where are attached 4 moieties (Fc, Ph1, Ph2 and Succ)) is partially inserted into a CD by one of its moieties. The methylation state of the CD was changed at each generation, inverting all Me and H (blue and green spheres, on wide and narrow side of the CD, the current modification for this generation is a red sphere). The best G1 model (other G1 models are not shown for clarity) was used to do the same for several generations. To show that the model is more stable, its insertion into the CD is drawn deeper (even though this is not so simple!). If the model is less stable (shallow insertion), this branch is finished (dead end, here in G3, the worse model is drawn specially for illustration) and a better model of the same generation is used to continue the tree, or from a previous generation.

The server has the XAMPP pack installed (Apache web server, PHP interpreter, MariaDB database server). The client (same computer or another one) communicates with the server by HTTP protocol. The server sends the dynamic webpage (content based on the PHP program and on the content of the database) to the client that displays it using its web browser (compatible with any OS, even if the server and the client are different computers that have different OS).

The image was created with Blender (up) and with Chemdraw (down) and was assembled with Paint.
